# Development of a Questionnaire and Cross-Sectional Survey of Patient eHealth Readiness and eHealth Inequalities

**DOI:** 10.2196/med20.2559

**Published:** 2013-09-02

**Authors:** Ray Jones

**Affiliations:** ^1^University of PlymouthFaculty of Health, Education, and SocietyPlymouthUnited Kingdom

**Keywords:** eHealth readiness, eHealth inequalities, digital divide, questionnaire development

## Abstract

**Background:**

Many speak of the digital divide, but variation in the opportunity of patients to use the Internet for health (patient eHealth readiness) is not a binary difference, rather a distribution influenced by personal capability, provision of services, support, and cost. Digital divisions in health have been addressed by various initiatives, but there was no comprehensive validated measure to know if they are effective that could be used in randomized controlled trials (RCTs) covering both non-Internet-users and the range of Internet-users.

**Objective:**

The aim of this study was to develop and validate a self-completed questionnaire and scoring system to assess patient eHealth readiness by examining the spread of scores and eHealth inequalities. The intended use of this questionnaire and scores is in RCTs of interventions aiming to improve patient eHealth readiness and reduce eHealth inequalities.

**Methods:**

Based on four factors identified from the literature, a self-completed questionnaire, using a pragmatic combination of factual and attitude questions, was drafted and piloted in three stages. This was followed by a final population-based, cross-sectional household survey of 344 people used to refine the scoring system.

**Results:**

The Patient eHealth Readiness Questionnaire (PERQ) includes questions used to calculate four subscores: patients’ perception of (1) provision, (2) their personal ability and confidence, (3) their interpersonal support, and (4) relative costs in using the Internet for health. These were combined into an overall PERQ score (0-9) which could be used in intervention studies. Reduction in standard deviation of the scores represents reduction in eHealth inequalities.

**Conclusions:**

PERQ appears acceptable for participants in British studies. The scores produced appear valid and will enable assessment of the effectiveness of interventions to improve patient eHealth readiness and reduce eHealth inequalities. Such methods need continued evolution and redevelopment for other environments. Full documentation and data have been published to allow others to develop the tool further.

## Introduction

### Definitions and Literature

The term eHealth is used in various ways, some (eg, World Health Organization) [1] using it as an over-arching term incorporating health informatics, telehealth, e-learning, and mHealth, while others think of eHealth as a patient-centered subset of health informatics [2-8]. This paper uses the term “patient eHealth readiness” to refer to the opportunity of patients to use the Internet and apps for health, and eHealth inequalities to mean differences in patient eHealth readiness.

In developing the ideas for this study, literature was first reviewed in November 2010 and updated in May 2013 from Web of Knowledge, PubMed, and Google Scholar using the terms (1) [E-health OR ehealth OR telehealth* OR telemedicine OR (ICT AND health) OR (technology AND health <in topic>) AND (readiness OR preparedness OR (implementation AND measure*) <in title>], (2) E-health literacy, (3) (Digital divide OR digital inclusion OR digital exclusion OR e-health inequalities) AND health, and (4) Inequality AND measure <in title> AND health. Web of Knowledge was used to examine citations of this literature for further relevant studies.

### Benefits From Patient eHealth Interventions

There is evidence that direct use of the Internet by patients can benefit patients [9]. For example, systematic reviews show improvements in health-related knowledge, attitudes, intentions and behaviors [10,11], and reduced health service use [12,13]. Studies showing the benefits of patient eHealth interventions, however, are nearly always carried out on populations of Internet users and the effectiveness of any intervention may depend on the skills and opportunities of the population recruited [10].

### Barriers to Patient eHealth Opportunity

There are four domains of barriers to eHealth opportunity that were identified (1) provision of eHealth opportunity, (2) personal abilities of the patient, (3) the support from others they may have to use eHealth, and (4) economic barriers.

Provision of eHealth opportunity varies. For example, while some British general practices [14] provided information, repeat prescribing, appointment booking, online advice, and patient access to their medical records, other practices had no website [15]. Internationally, many US practices use preconsultation computer-interviews [16], but these are rarely used in Britain. In secondary care, most British renal patients have access to their renal medical records online [17], but few stroke patients have such facility. Even use of globally available websites may show marked regional variation because of varied rates of recommendation to patients. For example, use of an online cognitive behavioral-therapy site for depression varied 30-fold by postcode area [18].

Physical and psychological attributes of patients contribute to digital divisions in health. Someone may have problems from sight or hearing impairment, arthritis, or lack of mobility in their hands. They may have no prior experience or find it difficult to learn Internet use, have limited literacy or health literacy, or lack confidence either in their Internet use or in making decisions using health information. They may distrust the Internet [19]. Someone’s current health may increase motivation to use the Internet for health [20], but may restrict Internet use; 81% of those with no recent health problems had used the Internet compared to 65% with recent health problems [21].

Some factors limiting personal use of eHealth may be diminished if people have support from others. For example, anonymous e-mail support may help people with long-term conditions use the Internet [22,23] and volunteers may help older people start using the Internet [24]. Without such support people may struggle to go online or make the best use of resources.

Finally, economic factors may affect digital divisions in health [25]. Although homes may be capable of Internet connection, families may not be able to afford it. Someone relying on accessing the Internet at their local library may be restricted by transport costs. Some groups, such as those with substance use problems, may be particularly susceptible, and in times of economic recession, barriers to eHealth use may increase. In the United States, broadband use is clearly related to income with 43% of families with incomes between $15,000 and $25,000 compared to 86% of those with incomes between $100,000 and $149,000 having home broadband [26]. However, with appropriate provision even the poorest can get access; a US study among homeless found that 47% reported computer use in the past month [27]. Economic factors are relative to the cost of alternative actions in health.

Others have examined barriers to eHealth use and the eHealth readiness of organizations or health services [28-51] through measures involving contact with staff or observation of process. The aim of this study was to develop a patient-completed tool giving patients’ perceptions of their opportunity that could be combined with their personal abilities, their support networks, and economic barriers.

### Do Digital Divisions in Health Deserve Action?

Should governments or health services address digital divisions in health? Some argue that it is just a matter of time before everyone has Internet access and that digital divisions will disappear. Others remind us that in the diffusion of technology [52,53], there are always earlier and later adopters, so there will always be inequalities. Others argue that as technology and eHealth progress, differences in opportunities for patients to use the Internet for health may increase, ultimately leading to worse health inequalities [54]. Even without the ethical argument for addressing inequality, eHealth inequalities make the adoption of more cost-effective health delivery difficult. If health services must provide eHealth and more traditional services, this diversity of service provision may be expensive. The digital divide has received attention with British government promoted organizations such as Race Online 2012, national regular reporting of digital use [55,56], and other specialized reports [57]. The current British government is committed to the idea that services should be “digital by default” [58], which may impact on those without good Internet access or skills.

### What Level of eHealth Inequality Is Important?

Like the seven-year difference in life expectancy by social class in England [59], the size of eHealth inequality needs to be large enough to be of concern. Some differences are binary; if houses in rural areas are not connected to the Internet, then those families cannot use eHealth. Other factors, such as eHealth literacy, will follow a distribution and we need to ask whether the standard deviation of that distribution is unacceptably large. In some cases relatively small differences are worth addressing if that can be done at low cost. A single numerical measure of eHealth inequality would help to judge the effectiveness of interventions.

### How Have eHealth Inequalities Been Addressed?

Initially, physical access to eHealth received a good deal of attention. From the late 1980s, there were experiments with public access kiosks [60] and initiatives to make the Internet available in public libraries. In the United States, 95% of public libraries provided Internet access by the year 2000 [61]. The third sector, through organizations such as Age UK, have provided both physical access and support using computers for older people [62]. In the English National Health Service (NHS), NHS Choices had a social and digital inclusion team from 2007 to 2012 [63], now lost in recent government cuts. There are no quality targets requiring NHS Trusts to provide eHealth services.

Various studies have addressed eHealth inequalities or tried to ameliorate their impact. For example, Kerr et al [64] explored the effectiveness of a web-based intervention in decreasing inequalities in access to self-management support in patients with coronary heart disease. Jones et al piloted anonymous personal online email support for patients with long-term conditions [22]. In the United States, an experiment offering older adults computer training in public libraries on finding health information via the Internet was successful [65]. In England, Fisher et al aim to improve uptake of patient access to their records by supporting general practices [66].

Digital divisions caused by physical disability have been subject to legislation. Web accessibility laws and regulations have encouraged developers to make websites accessible to those with visual, auditory, motor, neurological, or cognitive impairments. In Britain, the Disability Discrimination Act 1995 [67], Special Educational Needs and Disability Act 2001 [68], and the Equality Act 2010 [69] resulted in organizations reviewing website functionality and causing some organizations [eg, Royal National Institute for the Blind (RNIB)] [70] to have units aiming to make digital information accessible to those with physical disability. In the United States, section 508 of the Rehabilitation Act of 1973 [71] required federal agencies to ensure that federal employees with disabilities have equal access to information unless an undue burden would be imposed on the agency.

### Why Do We Need to Measure eHealth Inequalities?

Projects and national initiatives such as those described above need to measure eHealth inequalities to know (1) if action is needed, (2) what is the main cause of inequality, and (3) if inequalities are addressed, if the intervention was successful and cost effective. But inequality cannot be directly measured; it has to be measured as a difference in another variable, namely eHealth readiness. To compare eHealth inequality over time, we need a measure of “patient eHealth readiness” that is comprehensive, valid, and reliable. A measure that is also “diagnostic,” allows development of interventions tailored to the needs of populations.

### Tools to Assess eHealth Readiness

Others have considered the “readiness” of practitioners, organizations, or communities to adopt telehealth or eHealth [34,35,37,38,44-46,72]. Legare in 2010 [38] identified six eHealth readiness tools [28,29,31,32,34,44], five of which assessed organizational readiness. Legare developed one of these [34] further, translating it into French [37] and validated its use with staff. However, no suitable tool that assessed patients’ opportunities to participate in eHealth was identified.

There are two groups of literature that exist at the “patient level” (1) the “digital divide” and (2) eHealth literacy. The digital divide-as the term implies-tends to be measured as a binary division. For example, whether someone has or does not have access to the Internet or has or has not used the Internet in the last three months [73]. The digital divide has usually been assessed and reported by factual measures of Internet use or availability rather than attitudes or psychometric assessment. Work on measures of eHealth literacy [in particular the eHealth Literacy Scale (eHEALS)] [74] recognized that physical access to the Internet was only part of the story and that personal abilities to use the Internet were important. However, by adopting a more sophisticated examination of eHealth literacy, the basic ideas of digital divide and limitations of access to the Internet were lost.

Simple measures of whether or not someone has Internet access are insufficient as even among Internet users some may be more ready to make progress in using eHealth if they have access to support and are not struggling with the cost of access. In particular, interventions at patient and community levels need tools that can measure their impacts.

### Objectives

The aim of this project was to get the benefits of a scaled (rather than binary) approach (like eHEALS), but to include eHealth provision, support, and economics in the scale. In particular, the study aim was to develop and validate a self-completed questionnaire and scoring system for use in intervention studies hoping to improve eHealth readiness and reduce eHealth inequalities.

## Methods

### PERQ Stages

The Patient eHealth Readiness Questionnaire (PERQ) and related scores have been developed in two stages. First, four domains (1) provision (from the digital divide literature), (2) personal (from the eHealth literacy literature), (3) support, and (4) economic were used to draft a self-completed questionnaire and take it through three stages of piloting (January-March 2012). Second, a cross-sectional population survey was carried out (April-August 2012) and proposed scoring systems checked and iteratively refined to ensure construct validity.

### Physical

Although there are good national statistics on home Internet access [55,75,76] that allow a check on face validity, similar questions need to be included in patient-completed questionnaires to allow comparison before and after interventions. Provision of eHealth services is more difficult to assess as this will depend on the health conditions of interest to respondents and will be country-specific. Nearly all British respondents have a family doctor so asking about General Practice (GP) website provision is applicable to all. Some surveys have only asked about Internet “information” and respondents may not consider using the Internet to contact people. PERQ, therefore, included questions about personal contact.

### Personal

The most frequently used [77-80] measure of personal skills is eHEALS [74], using eight items to assess eHealth literacy. A Dutch translation of eHEALS was found reliable, but its validity questioned [81]. Van Deursen and Van Dijk [82] criticized eHEALS because respondents were not always accurate at estimating their real levels of skill [83]. Others have noted that self-efficacy may not accurately reflect ability. For example, nursing students’ self-efficacy in numeracy decreased if they had previously been asked to carry out an actual drug calculation [84], and patients with long-term conditions may be confident in what they do on the Internet, but lack a sense of adventure to try new things [22]. Van Deursen suggested that incorporation of basic Internet skills is needed to measure all aspects of eHealth literacy [85]. However, having to “test” whole populations to produce a measure of eHealth literacy is not feasible.

Prior to the Dutch studies [81,82,85], Hargittai [86] examined survey measures of Web-oriented digital literacy to serve as proxies for observed skill measures. They studied both observations and survey questions, and recommended measures as survey proxies of observed web-use skills. Their results suggested some composite variables of survey knowledge items were better predictors of people's actual digital literacy based on performance tests than the usual method of asking users' self-perceived abilities. Hargittai’s approach seemed a reasonable compromise towards the gold standard of Van Deursen. The first version of PERQ included the eight eHEALS questions and a single self-efficacy question, [22] based on [87], both “grounded” by using questions based on self-assessment (Hargittai’s approach) of the skills identified by Van Deursen and Van Dijk.

### Interpersonal and Economic Measures

Although interpersonal support to help people start using the Internet was a major component of the Race Online 2012 campaign [88], no “measure” of support in using the Internet was identified. Simple questions about who is available to support participants and if there are barriers (eg, of disclosure, or “being a bother”) can be used. Similarly, although economic differences in being able to use eHealth are clearly important, there did not appear to be any standard measures. To ground questions about the perception of cost of Internet access, PERQ included comparative questions about cost of access to health services and the perceived cost of Internet provision.

### Moderators

Whether or not someone uses the Internet for their health depends on whether they are motivated to do so [89]. So if patient eHealth readiness is to be an indicator of digital divisions, it needs to be “standardized” for motivation, similar to the distinction between digital choice and digital exclusion [90].

### PERQ Development and Initial Piloting

Three pilots (PERQ1-3) including repeatability were followed by a baseline survey. (PERQ4):

1. The first had 15 people (work colleagues and friends). Questions from eHEALS [74] were initially included following the four skills questions [85] and followed by a single self-efficacy question [22]. eHEALS score and the single self-efficacy rating showed quite good agreement (rho=0.61, *P*=.02), the single question showed good face validity (see Multimedia Appendix 1), and eHEALS was not well understood by one older person. Given the need to shorten the questionnaire, the eHEALS questions were subsequently omitted and the single self-efficacy question, following the four skills questions, retained.
2. The second had 20 friends and family of a research assistant; 17 of these were subsequently asked to complete PERQ4 to assess repeatability (reported below).
3. The third had a convenience sample of 103 houses likely to have a high proportion of more elderly residents. This was used to test the survey method, response rate, completeness of data, and that non-Internet users would respond. The response rate was 44% and data were reasonably complete. It was found that 5 out of 43 (12%) respondents had not used the Internet.

After each stage, revised questionnaires were circulated among colleagues to check readability (see Multimedia Appendix 1). The questionnaire was reviewed and approved by the university ethics committee between pilots two and three.

### Baseline Population Survey

As one intended use of PERQ was in a geographically based cluster randomized controlled trial (RCT), it was appropriate to test that mode of use. The aim was to use a sample representative of urban, suburban, and semirural postcodes and different levels of affluence to pilot the questionnaire, its distribution, and methods for construction of eHealth readiness and inequality scores. The 2001 census included 14,279 postcodes for the PL postcode area, with a total population of 510,223. There were seven postcodes (total population 3243) with very high populations (being either military camps or university halls of residence) that were excluded. The remaining postcodes were “ordered” by population and a 1% systematic sample (142 postcodes) was taken. Each postcode was “looked up” on the free Zoopla website service giving estimated property values to find current average property values and number of properties in each postcode. To achieve a “practical” sample of just fewer than 1000 properties, all postcodes further than 12 miles from Plymouth University were excluded, leaving 79 postcodes. These 79 postcodes were again ordered by Zoopla average property values, and a further systematic sample of 53/79 postcodes was taken with a total of 975 properties.

The final sample therefore comprised 53 postcodes within 12 miles of Plymouth University, with a total of 975 properties, population of 2126, and an average of 2.18 people per house. Average property prices (January 2012) per postcode ranged from British £78,163 to £459,360. The sample was representative of the range of property prices. Number of properties per postcode (a crude measure of rurality) varied from 1 to 53.

We attempted to deliver questionnaires by hand to all 975 properties in April 2012. There were thirty houses no longer in use, leaving 945 occupied houses as our sample. The research assistant called at each house and if someone answered, she explained the purpose of the survey and if possible handed the resident a questionnaire and covering letter (24 refused to take the questionnaire). If there was no response at the house, the questionnaire and covering letter were posted through the letterbox. In June 2012, reminders were posted through the letterbox of 658 who had not responded. The instructions on the questionnaire, and explained by the research assistant, were for the person with the next birthday in the house to complete the questionnaire.

### Analysis and Refinement of Scoring Systems

Survey data were used to assess the ability of PERQ to collect good quality data, and used to develop and iterate a scoring system suitable for use in intervention studies, particularly RCTs. This required that only questions that contributed to the score were included, collected data were complete, consistent, and valid, scales must not have floor or ceiling effects, and must reflect meaningful changes.

Scales that combine a number of “Likert style” attitude questions normally assess reliability using Cronbach alpha. In this study, the construction of the eHealth readiness scale relied on pragmatic combinations of factual questions with some ratings, so assessing scale properties such as repeatability, face, and construct validity, was also pragmatic. Questions were cross checked for consistency and reviewed for their contribution to eHealth readiness scores, face validation against other information sources, or description of sample demographics. Comparisons of scores between subgroups were made using Mann Whitney U tests for groups less than 100 and *t* tests for groups of 100 or more.

The contribution of each question was checked. Not all questions made direct contributions to scores, some were asked to ground respondents to give them the “right frame of mind” for subsequent questions. Other questions were used as consistency checks.

Scores for each constructed variable were essentially arbitrary, but to have some way of measuring change before and after interventions, an overall score is needed that is at least ordinal, and if possible approximates to a cardinal scale. Similarly, overall scores need to combine component variables in a sensible manner. A pragmatic and iterative approach was taken to examine construct validity of scores by examining the scores of sampled individual respondents with a range of scores. If the order and difference in scores between individuals did not match with an understanding of the barriers to adopting eHealth, the weights of scores were adjusted.

Scoring was also adjusted after examining the repeatability of scores and to cope with occasional missing values. This process of tuning scoring weights continued until all components seemed internally consistent. Various methods of combining the four subscales to produce an overall readiness scale were tried, checking for construct validity by examining differences between Internet users and non-Internet users.

### Questionnaire Review

Once the scoring system was finalized, questionnaires and dataset were again reviewed to check that all questions and answers were useful either as contributors to the score, as “grounding” for other questions, or as consistency checks (see Multimedia Appendix 1).

### Modeling of Performance in Measuring Change

Finally, data from the survey were used to model possible changes to participants “states” and to check the ability of the scoring system to measure those changes. Comparisons of scores between subgroups were made using Wilcoxon Signed Rank tests for groups less than 100 and *t* tests for groups of 100 or more.

This provided a further check that the subscores and weights seemed sensible, and to allow an assessment of methods of analysis and estimation of sample size for possible RCTs.

## Results

### Dataset

The anonymized dataset from the cross-sectional survey is available from the author.

### Response Rate and Possible Biases

**Figure 1:**
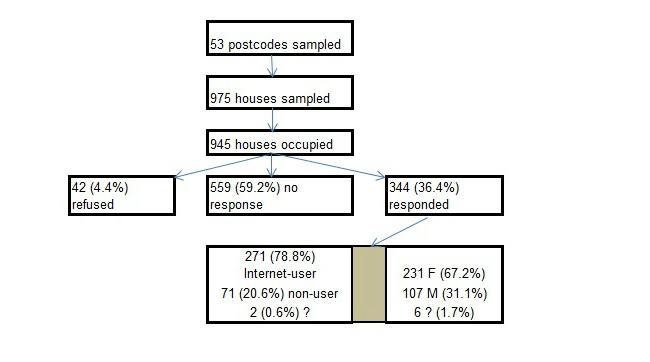
Sample response and characteristics.

Figure 1 shows by August 2012, 344 (36.4%) of the 945 occupied houses in the sample had returned completed questionnaires. Those 323/945 (34.2%) houses where the research assistant was able to speak to someone were more likely to have returned questionnaires (56% versus 27%) (χ^2^_4_=90.4; *P*<.001). The 344 houses providing respondents had higher estimated values than those with no respondent (£176,998 versus £142,019; *t*_925_=-6.2; *P*<.001).

### Data Completeness, Consistency, and Contribution

Despite care in design and three stages of piloting, PERQ still had missing and some inconsistent data (see Multimedia Appendix 1), for example 29/344 (8.4%) people did not complete their age and 6/344 (1.7%) their gender. All questions contributed (see Multimedia Appendix 1).

### Sample

Figure 1 shows the sample was disproportionately female (231/344 ,67.2% women; 107/344, 31.1% men; 6/344, 1.7% gender unknown), and older (mean age 55) than the Plymouth population.

### Constructed Variables

**Figure 2:**
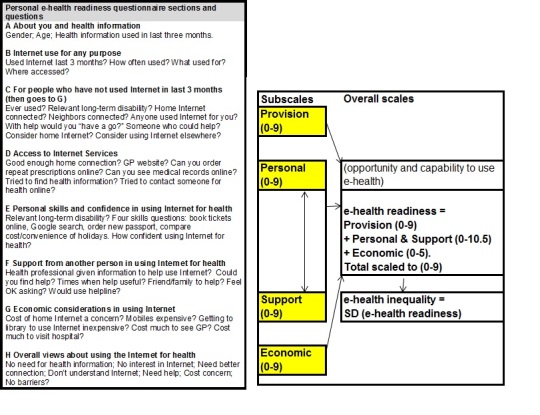
Personal eHealth readiness questionnaire and scale.

Figure 2 shows the eight sections of the questionnaire (A-H). Non-Internet users answered A, B, C, G, and H and Internet users A, B, and D-H. There were six sets of variables created by scoring or combining responses to questions (1) Need, (2) Internet-Use including range of uses and the four subscales of “eHealth readiness,” (3) Provision including physical provision of Internet and provision of health on Internet, (4) Personal (ie, the individual’s capability to use the Internet for health), (5) Interpersonal Support, and (6) Economic. A “short score” (of half the score) was used in some comparisons and figures.

### Need

Scores (0-10) representing “need for health information and support” were constructed from one multi-part factual question (A3), by adding 2 points for each professional contact and health-information seeking behavior in the last three months. Scores had face validity, for example, women had higher Need scores than men. Need was used as a modifier of Provision scores.

### Internet Use

Personal use of the Internet in the last three months was similar to national figures (271/342, 79.2% versus 77% from Office for National Statistics, ONS) [91]. As expected, younger people and those from more affluent areas were more likely to use the Internet. Nearly half used it for health (mainly to search for information), but few used discussion forums or social media for health purposes. Most Internet users (262/271, 96.7%) used the Internet at home, at work (94/271, 34.7%), or on mobile (93/271, 34.3%). This section (B) was used for subsequent questionnaire section choice, face validity check, and as a consistency check with other parts of the questionnaire.

### Provision

For Internet users, provision scores comprised two parts (section D) (1) General Internet Provision (4 points) ascertained by questions about what opportunities there are to access the Internet, and (2) Health Internet Provision (5 points) ascertained by questions on GP website (3.5 points), and Internet condition specific information and support (1.5 points). Provision of online information and support may vary by condition (eg, there are many resources for breast cancer, but fewer for stroke). However, not everyone has a need for health information or support and so may never have had reason to look for their GP website or for health information. So the Need score was used to moderate Provision; more was added to Health Internet Provision if Need was equal to zero (see Multimedia Appendix 1).

**Figure 3:**
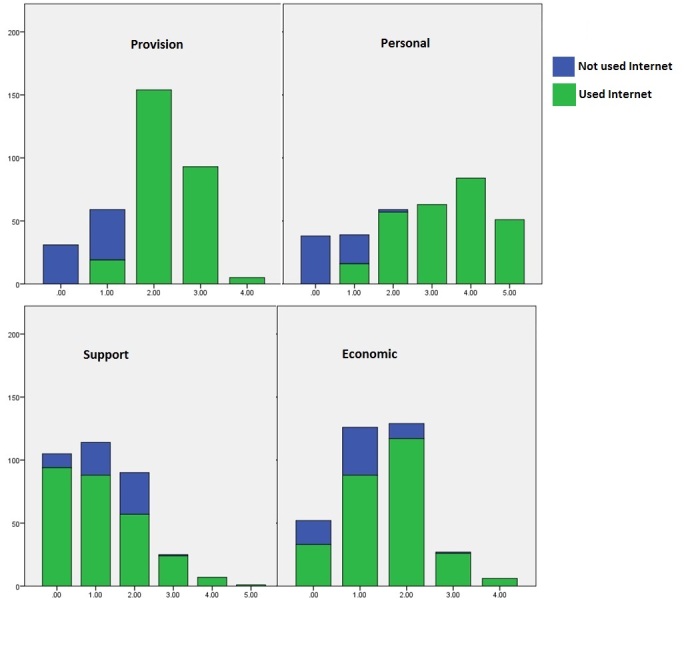
Four sub-scales of Provision, Personal, Support, and Economic presented as "short scales" of 0-5, showing Internet-users and non-Internet-users, including mean and standard deviation (SD) for full (0-9) scales.

Figure 3 shows the mean Provision score for Internet users was 4.5. There were 18 Internet users who had relatively low General Internet Provision (<1.5/3.5 max) including those who used the Internet only in places other than the home. Individual records were checked for participants with extreme scores and appeared to have face and construct validity (see Multimedia Appendix 1).

Component questions showed that nearly half (30/71) of non-Internet users had an Internet connected computer at home. Of 271 Internet users, 249 people had used it at home, but 3 said they had no home Internet connection, of these, 2 had used a mobile device and so it is possible that questions about “home Internet use” need to be clarified. There were 3 other people who had accessed the Internet at home, but did not apparently know if they had an Internet connection, may have not known about the “speed” of their home Internet, so some clarification may be needed for that question. A substantial minority (33/243,13.6%) thought their home Internet connection was not fast enough, a third of these said it was because they would need to pay more, a third because their provider did not offer a faster connection, and a third did not know.

Of 271 Internet users 89/271 (32.8%) had looked at their GP’s website, 51/271 (18.8%) thought their GP had a website, but had not seen it, 7/271 (2.6%) thought their GP did not have a website, and nearly half (122/271, 45.0%) did not know. Of the 89 who had looked at their GP’s website, 64 knew they could order repeat prescriptions online, 6 said their GP did not offer this service, and 14 did not know. Only 1 person knew they could see their medical record online, 20 knew they could not, but 66 did not know. Half (143/271, 52.8%) of Internet users had tried to find information on health topics with all but 9 having found what they wanted, but only 27/271 (10.0%) people had tried to contact an organization or forum or other people online connected with health.

The internal consistency of Provision scores was addressed by comparison of answers to sections B (where people had used the Internet) and D (home Internet provision and use for health). Figure 3 shows that no one had a short score of 5, so there was room for improvement and no ceiling effect.

### Personal

Personal scores comprised moderated confidence scores for Internet users (section E) and the willingness to try using the Internet for non-Internet users. Internet users rated their skills on four tasks from which skills scores (0-12) were constructed. Users then rated their overall Internet confidence (0-10). This sequence of questions aimed to ground their confidence rating in the reality of their ability and to provide a consistency check on their confidence rating. Skills scores correlated with confidence self-ratings (Spearman’s Correlation=0.60, *P*<.001) with some outliers; four people rated their skills low, but confidence high and 13 people rated their skills high, but confidence low (see Multimedia Appendix 1). However, to produce more consistent Personal scores, “moderated” confidence scores of skills*original confidence score/12 were calculated.

Component questions showed that just under half of non-Internet users said they would try using the Internet if they had help, would have a home Internet connection if they had help and it was cheap, and would use the Internet for health at some other place (most frequent choice public library). Questionnaires asked about disabilities. Six non-Internet users and six Internet users said they had disabilities (including arthritis, eye problems, hearing impairment, learning difficulty, and dyslexia) that made using computers difficult, but this information was not used in Personal score calculations on the assumption that respondents would themselves make that adjustment.

The face validity of Personal scores was assessed by exploring associations with frequency, range, and ubiquity of Internet use. As expected there were strong associations between frequency of use and moderated confidence (χ^2^_12_=81, *P*<.001). Nevertheless there were outliers, one person who was very confident despite using the Internet less than once a week and four people who used the Internet many times daily, but had low confidence. The latter is more believable as they may use it for limited purposes. Similarly, as would be expected, there were strong associations between range of use and moderated confidence (χ^2^_12_=61.5, *P*<.001), but similarly there were some “outliers.” Personal scores had a strong association with “ubiquity,” [ie, the places where people accessed the Internet (χ^2^_16_=81, *P*<.001)]. Some might argue that range of health uses should be the outcome measure of any intervention, but this will be dependent on someone’s need for health information. So overall PERQ scores include moderated confidence as Personal score, being a “cleaner concept,” but will additionally report range of health uses.

Figure 3 shows there were some ceiling effects on Personal score. Figure 3 also shows that despite grounding the estimates of confidence by asking about skills, there was still a large minority (51/200, 25.5%) of the sample with maximum scores, being able to do all four Internet tasks and being totally confident in their use of the Internet. This means that these people would not be able to increase their Personal score during the course of a study. This suggests that some “harder” tasks should be included in the skills question, and to focus the questionnaire better on eHealth, this should perhaps include some health-focused questions.

### Support

Support scores were largely based on factual questions. There were 22 out of 271 Internet users that did not complete the section on support, half of these (10/22) said (H1) that they had no barriers to Internet use and were confident in using the Internet for health (E3).

Component questions showed that among Internet users, only 58/271 (21.4%) had been given information by health professionals to help them use the Internet. Just under half (117/271, 43.2%) knew where they could find help locally in using the Internet; many of these (78) cited their local library. A quarter (68/243, 28.0%) said there had been times when help would have been useful, and of these, 50 had someone they could ask, of which 47/50 could ask about health. Nearly three-quarters (49/71, 73%) of non-Internet users had someone use the Internet for them. There were 40/71 (65%) that had someone that could help if they wanted to try using the Internet.

That Support scores were less differentiated between Internet users and non-Internet users “made sense” in the way that questions were asked and answered. Exploration of how Support and Personal scores were associated led to a pragmatic combination, using the Personal score to moderate the Support score in the overall Readiness score (see below). There were no ceiling effects on Support.

### Economic

The Economic subscale was constructed slightly differently to the other three subscales, relying on comparison of perceptions of the cost of using the Internet compared to other health activities such as visiting their GP or local hospital. Internet users and non-Internet users answered the same questions.

Considering the component questions, there were significant differences on the two Internet questions and on the cost of visiting the hospital between Internet users and non-Internet users. For the two Internet questions, this was dominated by the “don’t knows” among non-Internet users; 45% (29/64) of non-Internet users did not know about the cost of home access and 57% (35/61) about the cost of mobile access compared to 4.8% (13/269) and 32.8% (87/265) of Internet users. There was no difference between Internet users and non-Internet users in perceptions of cost in getting to the local library or GP. Most (195/325, 60.0%) did not think it cost much to get to a public library, but a large minority (95/325, 29.2%) did not know. The vast majority (317/337, 94.1%) agreed that visiting their GP cost nothing or very little. Non-Internet users were more likely to think that visiting their nearest hospital cost nothing or little (55/67, 82% versus 185/268, 69.0%; χ^2^_4_=15.7; *P*=.003); this may be because more had free bus passes and may be an important reason why the Internet appears relatively more expensive to older non-Internet users.

Figure 3 shows that overall, non-Internet users were likely to have lower Economic scores indicating more barriers to using the Internet (χ^2^_8_=39; *P*<.001). There were no ceiling effects so improvements could be measured.

### Overall View on Using the Internet for Health

Question H1 sought to identify the most important issue in using or not using the Internet for health. The original intention was to use this question to weight subscale scores in their combination to produce an overall readiness score. This idea was abandoned when it was realized that there was a close relationship between the Support and Personal subscales and an alternative combination method was developed. However, H1 remained a useful consistency check on the subscale scores. Table 1 shows that most Internet users (185/271, 70%) thought they had no real barriers to using the Internet for health. Among non-Internet users, 61% (40/66) said they had no interest in using the Internet.

Further breakdown of the groups in Table 1 show the range of different situations and attitudes. Of the 52 with “no interest in using the Internet,” 12 had used it in the last three months, 11 had home Internet access and had not used it personally, but most had someone else use it for them. Relatively few chose connectivity, economic reasons, or need for support as the main barrier to Internet use for health.

To test the construct validity of subscales, constructed variables were compared to answers to question H1 (Table 1). All but two answers, “would use Internet more if could get a better connection” and “would use Internet more if could get someone to help” showed significant differences on the expected variable. Short scores were compared between Internet users and non-Internet users and examples of where non-Internet users had higher scores or the same scores as Internet users were selected and reviewed. These showed construct validity.

### Combining the Four Subscales into an Overall eHealth Readiness Score

The initial intention was to create an overall eHealth readiness score by taking the mean of the four subscales, that is (Provision + Personal + Support + Economic)/4. However, exploration of the data led to recognition that Support was much more important for non-Internet users. Those who were already competent Internet users for health needed little support and scored low on Support. This reduced their overall eHealth readiness score and was misleading. Support was therefore added to eHealth readiness in inverse proportion to that person’s Personal score, (ie, people with a higher Personal score had less weight given to their Support score). Through a process of iteration considering whether the impact on overall eHealth readiness made sense, the term 3*Support/(Personal+Support) was added as a “Modified Support” term. This Modified Support score can range from 0 to 3 and the sum of Personal and Modified Support can range from 0 to 10.5. The Economic score also seemed less important in being “eHealth ready” than Personal and Provision scores, so the short score (range 0-5) was used as the contribution to eHealth readiness. So,

eHealth readiness= Provision (0-9) + (Personal + Modified Support) (0-10.5) + Short economic (0-5)

It was then multiplied by 9/24.5 to scale to the range 0-9. Figure 3 shows in this sample scores ranged from 0-7 with mean 4.1 (SD 1.79). Non-Internet users had, as expected, lower scores than Internet users.

**Table 1:** Numbers choosing statements (in section H) that best summarized their view of using the Internet for health and Mann Whitney *U* or *t* independent sample tests to assess correspondence between those statements and appropriate constructed scores. (15 missing values).

Overall View	Non-Internet user		Internet user	Total	“Nearest” variable	Mean score for those who chose this item versus rest (*t* test)	
	Home access	No home access					
						
(H11) No need for health information.	1	3	34	38	NEED	1.8 versus 3.3 *U*=3336, *P*<.001	
(H12) No interest in using the Internet.	11	29	12	52	PERSONAL	1.1 versus 6.2 *U*=774, *P*<.001	
(H13) Would use the Internet more for health if could get a good Internet connection.	0	2	3	5	PROVISION	2.8 versus 3.8 nsd	
(H14) Don’t understand the Internet that much.	9	4	17	30	PERSONAL	2.7 versus 5.7 *U*=1710, *P*<.001	
(H15) Would use the Internet more for health if could get someone to help.	0	0	10	10	SUPPORT	2.1 versus 2.1 nsd	
(H16) Would use the Internet more for health if money were no object.	1	2	2	5	ECONOMIC	0.8 versus 2.5 *U*=353, *P*=.024	
(H17) Uses or would use the Internet for health and have no real barriers to that use.	4	0	185	189	PERSONAL	7.2 versus 3.2 *t*=16.8, *P*<.001	
					ECONOMIC	3.0 versus 1.9 *t*=6.3, *P*<.001	
					PROVISION	4.0 versus 1.9 *t*=13.7, *P*<.001	
					SUPPORT	3.1 versus 2.3 *t*=4.6, *P*<.001	
					READINESS	4.4 versus 2.3 *t*=16.1, *P*<.001	
Total	26	40	263	329			

^a^ *U*=Mann Whitney *U*

^b^nsd=no statistically significant difference *P*>.05

### Repeatability and Change of Scores Over Time

Seventeen of the 20 people who completed the second-stage pilot (January 2012) completed PERQ4 in September 2012. Of these, four non-Internet users were excluded as the questions in section C had changed too much between the earlier and later version of PERQ to be comparable. For 13 Internet users their January data was converted to the September version of the questionnaire to allow a comparison and some assessment of “repeatability” and change over time. Each pair of questionnaires was examined for changes to answers and the impact on the scoring system to see if it made sense and if the scoring system was appropriate. This check resulted in some changes to the scoring system. With the final scoring system there was reasonable consistency in scores between January and September 2012 (see Multimedia Appendix 1) with changes in scores making sense with known changes in personal circumstances for those respondents.

### Is PERQ Suitable to Assess Interventions?

**Figure 4:**
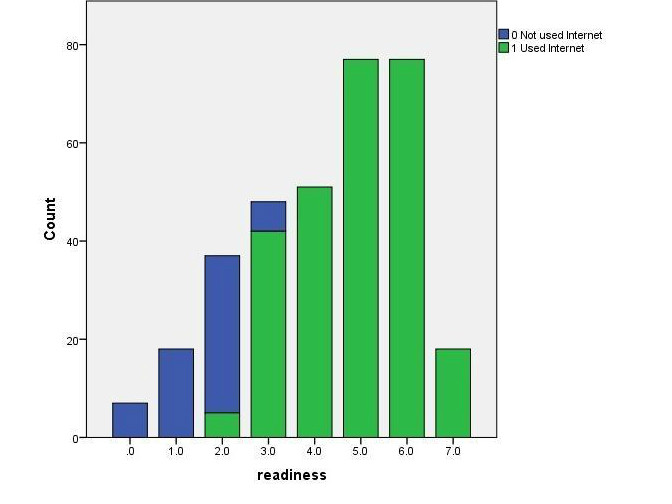
Distribution of eHealth readiness scores on possible scale 0-9 showing Internet-users and non-Internet-users.

Figure 4 shows that PERQ did not have floor or ceiling effects. PERQ produces two scores, eHealth readiness and eHealth inequalities (SD of readiness). The aim of interventions would be to improve overall eHealth readiness (ie, increase the mean score) while keeping variation (SD) the same or reduced. With this sample, the mean eHealth readiness score was 4.24 with standard deviation 1.73, (4.9 for Internet users versus 1.6 for non-Internet users; *t*_4.24,1.73_=-25.8; *P*<.001). Statistically significant changes in mean scores must represent practically (clinically) significant changes. To assess whether this scoring system can measure an attainable and useful improvement in eHealth readiness and what this would mean in terms of individual changes, changes were modelled using the dataset.

Table 2 shows four feasible changes resulting from interventions or further development in Internet use, modelled using the dataset. The first shows that if 20 non-Internet users get online and access health information there is a substantial increase in score for subgroup and whole sample as well as a reduction in inequality (SD of readiness). While the decrease in SD is not statistically significant (confidence intervals are 1.73-6.38 and 1.50-5.53) [92] the decrease is at least “heading in the right direction.” The second shows the impact of existing Internet users gaining more routes to access via mobile and learning about patient access to their GP record. Mean readiness is increased, but again, although not statistically significant, it is tending to increase inequality. The third scenario might result from better Internet provision, such as the implementation of faster broadband as is happening in Cornwall. If the level of statistical significance is set at *P*=.05, then the increase in readiness is significant, but policy makers may consider the actual change of just 20 people getting faster access relatively unimportant. With this sample size it appears relatively easy to achieve a statistically significant change in mean PERQ. The fourth scenario shows the possible impact of GPs in the area starting to engage more in recommending websites to their patients, as has been the case with “information prescription” projects [93-99]. PERQ therefore appears to be sufficiently sensitive to change.

**Table 2:** Modelled results of interventions, showing impact on subgroup and whole sample on eHealth readiness score and eHealth inequality and Wilcoxon signed ranks test (*z* statistic) or paired *t* test with original data.

Assumed changes	Impact on sub group mean readiness score	Impact on whole sample	
		Mean readiness score	Inequality (standard deviation of readiness)
			
20 non-Internet users are supported in getting online. They have not looked at the GP website, but have found other health information online.	Increase 1.8 to 5.0 *z*=4.0; *P*<.001 n=20	Increase 4.24 to 4.44 *t*=4.48; *P*<.001 n=333	Decrease 1.73 to 1.61
20 Internet users who previously used computer at home and at work got mobile access and became aware of GP services including patient access to records.	Increase 4.9 to 6.2 *z*=4.1; *P*<.001 n=20	Increase 4.24 to 4.32 *t*=4.34; *P*<.001 n=333	Increase 1.73 to 1.79
20 Internet users who said their Internet connection was too slow who got a faster connection and many of who used it to contact someone about health.	Increase 4.2 to 4.6 *z*=2.8; *P*=.005 n=20	Increase 4.24 to 4.27 *t*=2.86; *P*=.005 n=333	No change 1.73
80 Internet users who previously had not had advice on using Internet from HCP, recommended websites by GP.	Increase 4.5 to 4.7 *z*=4.0; *P*<.001 n=80	Increase 4.24 to 4.29 *t*=4.09; *P*<.001 n=333	No change 1.73

## Discussion

### Questionnaire and Scoring

A questionnaire (PERQ) and scoring system has been developed comprising four components of patient eHealth readiness (1) provision of Internet and Internet for health, (2) personal capacity to use it, (3) support in using it, and (4) economic barriers to use. The scoring system appears consistent, to have face and construct validity, and to produce a score that can be used to assess interventions that improve eHealth readiness. By examining the standard deviation of scores, eHealth inequalities can be reviewed to ensure that interventions have not worsened inequalities. The questionnaire is being used in two studies locally [24,100].

Although national cross-sectional data from the ONS and Oxford Internet Survey (OIS) show the uptake of the Internet, and sometimes include questions on health, there is a need for a tool to measure the impact of interventions in the context of RCTs. Although there are widely used measures of eHealth literacy, these were not sufficiently comprehensive in their scope; in particular they only “worked” for Internet users. Initially the eHEALS questionnaire on eHealth literacy was included within the PERQ questionnaire, but was then dropped as the single self-efficacy question seemed adequate and took less space. There was a need for a measure that covered the full range of individuals from non-Internet users through to frequent Internet users.

### Limitations in Scoring System

This pragmatic scoring system has many limitations, but is published with full details and data so that others can refine or continue to develop it. One problem with any measure of eHealth readiness is continually changing technology such as the current shift to smart phones [101]. If measures are to be used for any length of time they need to cope with changing technology. One solution may be to have a framework of generic questions that remain the same over time, but “situate” these by inserting questions related to the “state of the technology.” These questions will change over time as technology changes. The first stage of using a measure of eHealth inequality for a study would be to gain consensus on the current “State of Technology.” This has not been achieved in the development of this questionnaire and scoring system, but remains a future goal.

Second, the scale and scoring rely on self-report. Although one dimension of the proposed scale is provision of eHealth services, and although this could be measured fairly objectively [15,18], to have a method consistent with the other dimensions, this is best measured by asking patients (ie, the pragmatic solution of “perception of provision.)” Self-reported measures, such as self-efficacy, as discussed earlier, may not be good predictors of actual ability. PERQ tries to compensate by using “grounding” questions and by pragmatic “constructs” such as “modified confidence.”

Third, having four dimensions makes having one summative number for eHealth readiness difficult. As described, the original plan to make the four components additive either in equal proportion or, using the ideas of Paterson et al [102-105] in measures of quality of life, by asking respondents to nominate what is most important. However, when the close connection between the Personal and Support terms was noted, this determined how the four terms should be combined. The final scoring system appears to have face and construct validity, but is nevertheless arbitrary. Others may wish to explore alternatives.

Fourth, the eHealth readiness scale is at best ordinal and not cardinal. This compares to, for example, a difference in mortality that can be expressed as a difference in years of life. While some may argue that 10 years of life at age 20 is “worth more” than 10 years of life at age 70, “years of life” is essentially a cardinal scale. Self-reported questions used to construct an ordinal scale will always have limitations and should be used cautiously.

Fifth, the weights used for individual items were arbitrary. For example, Internet Health Provision included questions about whether patients could access their medical records (weighted 1.5) and could order repeat prescriptions online (weighted 1.0). These weights reflect the judgements of the author in the “difficulty” or “sophistication” of provision. Clearly other weights could be used and the dataset and analysis syntax are provided for others to explore, but this pragmatic approach seems to provide a way of scoring and measuring change.

Sixth, people whose opportunities to use the Internet are less because of limited English will not be identified by this approach, using an English language questionnaire.

Lastly, it is not possible to directly measure eHealth inequalities, but instead to examine the spread of eHealth readiness scores. Those implementing eHealth interventions are interested in improvements in mean score (eHealth readiness) over time, but should also consider the standard deviation of scores (eHealth inequality). In other fields, for example, income inequality, measures such as the Gini coefficient directly measure inequality. Health inequalities are typically measured by differences in mortality, expressed in absolute numbers of life expectancy at birth or some other age between two groups. In this case, we hope that an intervention will improve eHealth readiness, but also reduce the standard deviation. It was important therefore in the construction of this score that a reduction in standard deviation was not artificially induced by a “ceiling effect” on the score. Given the natural progression of the Internet we are unlikely to see eHealth readiness reduce, so “floor effects” are less important. The modelling of possible interventions suggests that the eHealth readiness score is quite sensitive to relatively modest changes in Internet use for health. In determining sample size and setting significance levels, therefore, it is suggested that *P*<.001 is appropriate. On the other hand, achieving a statistically significant reduction in eHealth inequality may be difficult, but researchers’ and policy makers’ may be able to decide that interventions are at least not making inequality worse.

### Social Determinants

Clarity is needed about the role of social determinants of eHealth inequalities. Should associations between eHealth use and demographic and social variables be explored, or should the focus be on the immediate “cause” of eHealth inequality? Demographics are clearly important in use of the Internet [76]. Answers to questions on the personal and interpersonal components of a measure may be predicted by social determinants and act as a test of face validity, but should not be part of any eHealth readiness scoring system.

### Representativeness of the Sample

That the nonresponders in the baseline survey were more likely to live in lower value houses will have biased this sample towards Internet users [76]. On the other hand, using households as the sampling unit biased the sample towards older people and females (as there are more single, older, female households), so biasing the sample towards non-Internet users. Lower response rates from younger people, particularly from student households, will also bias the sample towards older people and non-Internet users. Overall, the baseline survey overrepresented older people. As the purpose of the sampling was to have a “test bed” for the questionnaire and to develop the scoring system, this may have been an advantage rather than disadvantage. The selection of households and respondents for this survey was pragmatic using easily available open data sources, but was similar in principle to the methods used in the OIS. The OIS used 175 randomly selected “Output Areas” in England, within which 10 addresses were selected at random from the Postal Address File. Interviews aimed to interview the person with the next birthday.

The baseline survey response rate was fairly poor (36%) compared to the 59% achieved by ONS [55] and to the OIS (49% successful interviews for 4160 houses visited) [76]. But, with bigger budgets, the data for both were collected by interview (rather than returned self-completed questionnaire) and allowed multiple visits to find a respondent at home. In this survey, the response rate for houses where the research assistant was able to speak to the resident before leaving a questionnaire for self-completion and return was 56%. Greater variation in time of calling/delivery and a budget allowing more persistence should achieve a better response rate.

### “Diagnostic” Uses of PERQ

More detailed analysis of PERQ results could indicate the most appropriate interventions for individuals or subgroups. For example, groups that would most benefit from faster access, or support, or for whom economics was the main barrier could have interventions chosen appropriately. It is possible that a “stages of change” approach to classifying individuals might be useful, although the different dimensions (personal, provision, economic, support) need to be taken into account.

### Further Work

The support section of the questionnaire was the least successful. This had proved difficult throughout piloting. In particular, we had sought ways of getting those people who had never needed or sought help to answer the questions by wording the questions about “people in general,” and by stressing that we wanted everyone to answer this section. Nevertheless, 21/271 (7.8%) Internet users failed to answer this section. The second part of the “support section” (F) of PERQ probably did not collect particularly useful information, and given the desire to shorten the questionnaire, could possibly be dropped in further developments.

### Conclusions

There was previously no measure of personal eHealth readiness or eHealth inequalities. The concept of a patient eHealth readiness based on provision, personal ability, support, and economic considerations with eHealth inequality as the standard deviation seems to “work” and be acceptable in a British context. The scores produced appear valid and sufficiently sensitive to enable assessment of the effectiveness of interventions to improve eHealth readiness and reduce eHealth inequalities. With suggested modifications PERQ is now being used in two other local studies. It could also be used to help identify interventions addressing eHealth readiness. Such methods need continued evolution; full documentation and data have been published to allow others to develop the tool further. In particular with changes to the “provision section,” PERQ might be adapted for use in nonBritish settings.

## Multimedia Appendix 1

Initial piloting, more details on baseline survey, questionnaire development (versions 1-4), variables, SPSS syntax, dealing with inconsistent and missing data.

## Abbreviations

GP: General Practice
OIS: Oxford Internet Survey
ONS: Office for National Statistics
PERQ: Patient eHealth Readiness Questionnaire
RCT: randomized controlled trial
