# Supplementary material for: Development of a Questionnaire and Cross-Sectional Survey of Patient eHealth Readiness and eHealth Inequalities
Source: Med 2 0. 2013 Sep 2;2(2):e9. doi: 10.2196/med20.2559 (PMC4084763; doi:10.2196/med20.2559)
Supplement: Supplementary file 1 [file med20_v2i2e9_app1.pdf]

Appendix

Development of a questionnaire and cross-sectional survey of personal e-health readiness and e-health inequalities

		Page
1.	**INITIAL PILOTING OF PERQ**	2
2.	**BASELINES POPULATION SURVEY TO PILOT PERQ AND ITS DISTRIBUTION**	4
	2.1 Methods	
	Sampling of addresses	
	Final sample	
	Delivery of questionnaires	
	Analysis	
	2.2 Results	
	Response rate and possible biases	
	Data completeness and consistency	
	Need for questions	
	Sample	
	Constructed variables: Need, Internet Use, Provision, Personal, Support, Economic, Readiness	
	Overall view on using Internet for health	
	Face validity of short scores	
	Face validity of e-health readiness score	
	Repeatability	
	Using PERQ to assess interventions	
	Diagnostic uses of PERQ	
3.	**QUESTIONNAIRE DEVELOPMENT**	23
	PERQ1	24
	PERQ2	32
	PERQ3	39
	PERQ4 (final version in this study)	46
4.	**VARIABLES FROM PERQ4 QUESTIONNAIRE AND THEIR USES**	53
5.	**SPSS SYNTAX FILE FOR PERQ4**	54
6.	**DEALING WITH INCONSISTENT AND MISSING DATA**	60
7.	**REFERENCES**	61

1. INITIAL PILOTING OF PERQ

Pilot questionnaires
Section 3 includes copies of PERQ at each stage showing its evolution.

First stage pilot amongst 15 people

One major decision in the first stage of piloting was to drop the use of eHEALS. This explains the rationale for so doing.

In the first stage (PERQ1) we included questions from the eHEALS questionnaire following the 4 ‘Van Deursen’ questions and followed by a single self-efficacy question. The questionnaire was piloted with 15 people: 8 work colleagues and 7 family and friends (1 young male and 6 people from retirement apartments) who completed the questionnaire and talked about the questions with the researcher. One 86-year old woman, although she completed the questionnaire commented that she did not understand the eHEALS questions and her answers were (in her view) arbitrary.

Visual inspection of a scattergram (Figure 1) of eHEALS score and the single self-efficacy rating showed quite good agreement, confirmed by rank correlation. (Spearman rho = 0.61, p=0.02) Two outliers (with higher eHEALS scores) seemed to rate themselves too highly on eHEALS compared to other answers.

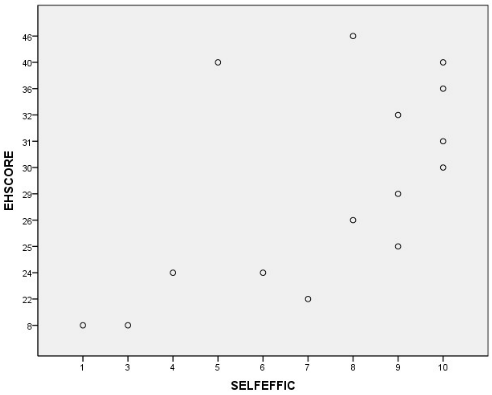

Figure 1. Single self-efficacy score Vs eHEALS score

You would expect some correlation between number of Internet uses and e-health confidence as a measure of face validity. The single self-efficacy question seemed to show some agreement (Figure 2) with the number of Internet uses (rho=0.55, p=0.03), whereas the eHEALS question did not (rho=1, p=0.5).
.

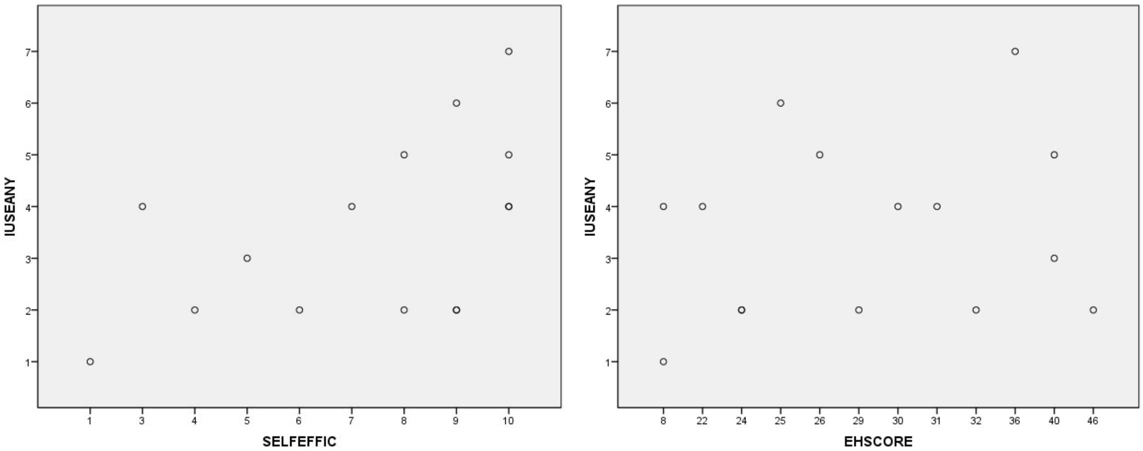

Figure 2. Number of Internet uses Vs single self-efficacy score (left) and eHEALS (right)

You would expect some agreement (face validity) between the ‘Van Deursen score’ (people’s perception of how well they could carry out certain Internet tasks) and their self-efficacy in using the Internet. Both eHEALS and the single self-efficacy score showed agreement with Van Deursen (Figure 2a) but this was stronger for the single self-efficacy question (rho=0.83, p<0.001) than for eHEALS (rho=0.55, p=0.03).

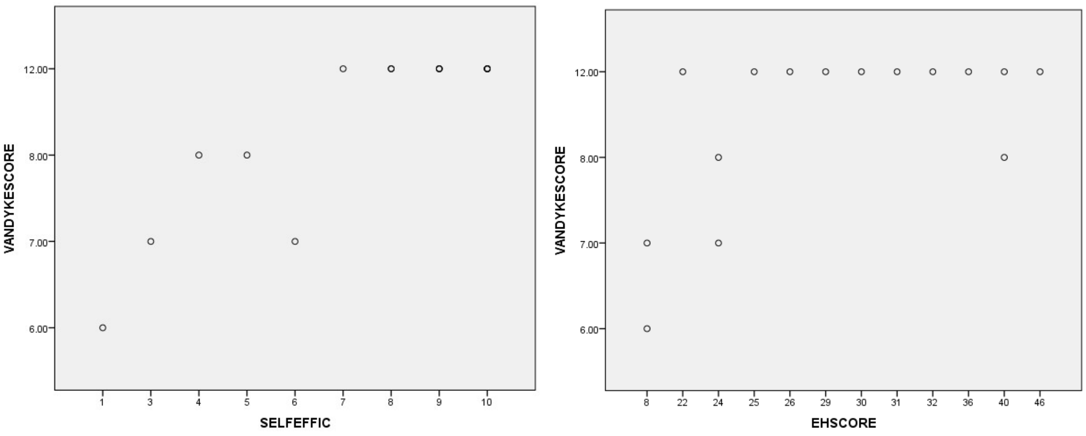

Figure 2a Scattergrams of self-efficacy and eHEALS against Van Deursen scores

Given that the PERQ was already quite (probably too) long, and that the eHEALS took 8 questions to complete rather than one simple self-efficacy question, and that the single self-efficacy question seemed at least as good as eHEALS, we dropped the eHEALS questions and just retained the single self-efficacy question, following the four Van Deursen questions in the questionnaire.

Second stage pilot amongst 20 people

Twenty friends and family of the research assistant were recruited (PERQ2) (January 2012) and asked about ease of questionnaire completion and any remaining ambiguities. Seventeen of these were subsequently asked to complete PERQ4 to assess repeatability.

Third stage pilot: convenience sample of 103 houses in two postcodes

Ethical approval for this and subsequent stages was obtained from the Faculty of Health, Education, and Society and the University of Plymouth. A convenience sample, of two areas (103 houses) in north Plymouth that were likely to have a high proportion of more elderly residents, and was not part of the sample for the baseline survey, was chosen. We particularly were concerned that people who did not use the Internet would not respond and wished to check responses. This stage of piloting (PERQ3) was used to check the response rate using our method of household delivery, completeness of data from self-completion and postal response, and to start piloting the SPSS syntax file and data checking. Delivery was attempted to 103 houses, 5 were empty and one person refused the questionnaire. After one reminder we obtained 43 responses (43/98 (44%) occupied houses). Five out of 43 (12%) had not used the Internet and had completed the relevant section of the questionnaire.

2. BASELINE POPULATION SURVEY TO PILOT PERQ AND ITS DISTRIBUTION

2.1 METHODS

Sampling of addresses

The 2001 census included 14,279 postcodes for the PL postcode area, with a total population of 510,223. Seven postcodes (total population 3243) with very high populations (being either military camps or university halls of residence) were excluded. The remaining postcodes were ‘ordered’ by population, and a 1% systematic sample (142 postcodes) was taken. Each postcode was ‘looked up’ on a free website service giving estimated property values (www.Zoopla.com) to find the current average property value and the number of properties in that postcode. If Zoopla did not have an average property value we used the next postcode in the postcode list as a replacement. (This may have led to the sample being biased towards urban and suburban housing as missing Zoopla values were more likely to be in rural areas).To achieve a ‘practical’ sample of just under 1000 properties we excluded all postcodes further than 12 miles from University of Plymouth (Figure 3), leaving 79 postcodes. (This may have further biased our sample towards urban and suburban housing.) The 79 postcodes were again ordered by Zoopla average property value and a further systematic sample of 53/79 postcodes was taken with a total of 975 properties.

Final sample

The final sample therefore comprised 53 postcodes within 12 miles of the University of Plymouth, with a total of 975 properties and (2001 census) population of 2126, with an average of 2.18 people per house. The average property prices (January 2012) per postcode ranged from £78,163 to £459,360. The sample was representative of the range of property prices. The number of properties per postcode (a crude measure of rurality) varied from 1 to 53.

Delivery of questionnaires

We attempted to deliver questionnaires by hand to each of the 975 properties in April 2012. Thirty houses no longer existed, were empty, or had become commercial properties, leaving 945 occupied houses as our sample. The research assistant called at each house and if someone answered, she explained the purpose of the survey and if possible handed the resident a questionnaire and covering letter (24 refused to take the questionnaire). If there was no response the questionnaire and covering letter were posted through the letterbox. In June 2012, reminders were posted through the letterbox of 658 who had not responded. The instructions on the questionnaire, and explained by the research assistant, were for the person with the next birthday in the house to complete the questionnaire.

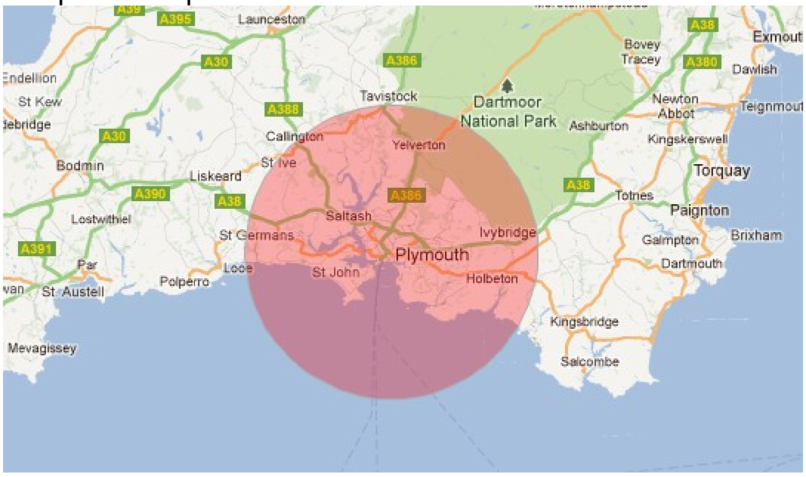

Figure 3. Location of survey sample: twelve mile radius from Plymouth

Analysis
The scores for each of the constructed variables are essentially arbitrary but to have some way of measuring change before/after in an RCT we need some overall score that is at least ordinal, and if possible approximates to a 'cardinal scale', i.e. we needed scores for each of the component variables where (e.g.) a score of 5 was 'better' than a score of 4, regardless of how the score had been composed. Similarly we wanted to construct an overall score in which the component variables are combined in a 'sensible manner'. A pragmatic and iterative approach has been taken to examine the 'face validity' of scores by examining the scores of sampled individual respondents with a range of scores, and if the order and difference in scores between individuals did not match with an understanding of the barriers to adopting e-health, the weights of scores were adjusted. Scoring was also adjusted after examining the repeatability of scores and to cope as far as possible with occasional missing values. This process of 'tuning' the scoring weights continued until each of the separate components seemed 'internally consistent'.

Various methods of combining the four subscales to produce an overall readiness scale were then tried, checking for face validity by examining differences between Internet and non-Internet users. Initially, one of the questions (H1 (see section 3)) was used to weight the most important component in the calculation of the overall e-health readiness score and weighted each sub-component the same (0-9). However, it became clear that the personal and support components interacted and so should be scored to allow for that interaction. The use of H1 to weight sub-components was then dropped, but it was still used as another construct validity check.

Finally, changes to the answers to various questions on PERQ for various sub-groups were modeled to estimate the effect of interventions on both sub-scores and the overall readiness score. This provided a further check that the sub scores and weights seemed 'sensible', and to allow an assessment of methods of analysis and consideration of sample size and significance level for possible randomised controlled trials.

2.2 RESULTS

Response rate and possible biases
By the end of July 2012, of the 945 occupied houses in the sample 344 (36.4%) had returned completed questionnaires, 559 (59.2%) had not responded, and 18 (3.6%) had sent back empty questionnaires. Including residents who had refused to take a questionnaire we had a total of 42 (4.4%) active refusals.

Those 323 where the research assistant was able to speak to someone, and they did not refuse, were more likely to be returned compared with houses where the questionnaire was posted (56% Vs 27%) (χ^2=90.4; 4df; p<0.001). There was no difference in the age or gender of those who responded having been spoken to, and those who responded with no conversation.

Of the 945 occupied houses, the 344 that provided respondents had higher 'Zoopla' values than those from which we got no response (£176,998 Vs £142,019; t=-6.2; 925df; p<0.001). There was no difference in Zoopla house values between those who responded and those who refused. Response rate by postcode varied from 0 to 100%.

Data completeness and consistency
Despite care in the design, piloting, and development, PERQ still has missing and apparently inconsistent data. Twenty nine (8.4%) people did not complete their age and 6 their gender; 312/344 (91%) completed both. Two people only completed one or two questions in section A; 342/344 completed enough to assess whether they had used the Internet.

Later in this Appendix there is an SPSS syntax file of more detailed 'cross-checks' between different parts of the questionnaire. In some cases it is possible to infer the values of missing data and 'correct' some of the inconsistencies. The SPSS syntax file creates variables called QUERY with values as shown later in this Appendix.

In some cases responses have been 'averaged'. Questions D6 and D7 should have cross-checked with B3 – use of the Internet for health. Discrepancies between the two show how people may misinterpret or misremember. Table 1 shows that 13 people answered B3 as if they had searched for health information but not D6, and 29 vice versa. The nine people who (D6) had tried to find health information but not found it show the possible ambiguity in B3. Nevertheless there was (109+105/256) 84% agreement between the two questions. 'Finding health

information' contributes to 'Provision', so an 'optimistic' view was used and if either B3a2=1 or D6=2 it contributed to 'Provision'.

B3 used the Internet for health related	D6 tried to find health information			Total
	Never tried	Tried and found	Tried & not found	
Not ticked	109	29	5	143
Find health information	13	105	4	122
Total	122	134	9	265

Table 1. Comparison of answers to B3 and D6 for 265 who had used the Internet.

Similarly, Table 2 shows that 20 people answered B3 as if they had used the Internet to communicate about health but not D7, and 17 vice versa. Agreement was 228/265 = 86%.

B3 used the Internet for health related comms	D7 tried to communicate for health			Total
	Never tried	Tried and found	Tried & not found	
Not ticked	222	17	3	242
Find health information	20	6	1	27
Total	242	23	4	269

Table 2. Comparison of answers to B3 and D7 for 269 who had used the Internet.

24/271 people did not complete section F even though they had used the Internet. This suggests that the wording of section F could be improved.

Need for questions

Each question was reviewed for its contribution to the e-health readiness scores, validation against other information sources or description of sample demographics, and cross-checking within the questionnaire. Some questions are asked to ground the respondent to give them the 'right frame of mind' for subsequent questions. The Appendix shows how all variables were used. Some questions may not provide sufficient information or utility to warrant their continuation in the questionnaire: currently the question on disability (E1) and the open question (H2) have not received much 'use' in this report but perhaps just needs more thought on analysis.

Sample

The sample was disproportionately female, comprising 231 (67.2%) females and 107 (31.1%) males, (6 gender unknown). The sample was also older than the Plymouth population; mean age (based on 312/344) was 55. The women were younger than the men (53 Vs 59; t=2.49; 310 df; p=0.013) (Table 3).

	Plymouth		PERQ sample	
Age	Males	Females	Males	Females
All Ages	94900 (100%)	93300 (100%)	96 (100%)	216 (100%)
15-29	37100 (39.1%)	32500 (34.8%)	9 (9.4%)	21 (9.7%)
30-49	31000 (32.7%)	32300 (34.6%)	18 (18.8%)	72 (33.3%)
50-59	14100 (14.9%)	15100 (16.2%)	16 (16.7%)	38 (17.6%)
60-69	12700 (13.4%)	13400 (14.4%)	26 (27.1%)	42 (19.4%)
70+	12100 (12.8%)	17600 (18.9%)	27 (28.1%)	43 (19.9%)

Table 3. Age and gender for the 312/338 who answered both questions compared to the adult population of Plymouth (http://www.plymouth.gov.uk/population)

Constructed variables

Six main sets of variables were created by scoring or combining the questionnaire responses: (i) need (0-10), (ii) internet use including range of uses, and the four components of 'e-health readiness' each scored from zero to nine (iii) provision including provision of internet and provision of health on internet, (iv) personal (capability), (v) (inter-personal) support, and (vi) economic. The weights and methods of combination were iteratively revised and refined as the data were explored to ensure a consistency within the subscales. The final versions of those subscales are presented in the following sections. The combination of the subscales into an overall 'readiness' scale, is described later.

Need

A score (0-10) representing 'need for health information and support' was constructed from a factual question, by adding 2 points for each professional contact, health information seeking behaviour in the last 3 months (question A3) (Table 4). Table 5 shows the distribution of 'need' scores. We also calculated a 'non-Internet' need score (0-8) by dropping question A3e. Non Internet users were slightly more likely to have visited the doctor in the last 3 months 85% Vs 72%, (χ^2=4.28; 1df; p=-0.04). (This is no surprise as non Internet users are older).

Women had higher mean 'need' score than males (3.4 Vs 2.7; t=-2.72; 327 df; p=0.007). There was no difference in 'non-Internet' need between those who had or had not used Internet in last 3 months but those who had used the Internet had higher 'need'. Just under half (42%) of Internet users had used it 'for health' in the last three months. The paper describes how 'need' is used to modify the provision score. It was debatable as to whether it was more appropriate to use need (including use of Internet) or non Internet need, but the full need score seemed the most appropriate.

Analysing individual questions, non-Internet-users were slightly more likely to have visited the doctor in the last three months 85% Vs 72%, (χ^2=4.28; 1df; p=0.04). This is not surprising as non-Internet-users are older. Just under half (42%) of Internet-users had used the Internet for health in the last three months.

Type of contact/information	Not used Internet N (%)	Used Internet N (%)	All N (%)
Seen a doctor, nurse or other health professional	57 (85%)	191 (72%)	248 (72.4)
Asked a family member or friend	14 (21%)	72 (27%)	86 (25.3)
Phoned a helpline	2 (3%)	13 (5%)	15 (4.7)
Read a book or magazine	11 (16%)	47 (18%)	58 (16.6)
Used the Internet	0 (0%)	112 (42%)	112 (32.8)
None of the above	8 (12%)	51 (19%)	59 (17.2)
Total	67	266	333 (100%)

Table 4. Number (percentage) who sought information, or advice, in the last three months about health for 333 respondents (11 missing values).

Score	Need		Need non Internet	
	Not used Internet	Used Internet	Not used Internet	Used Internet
**0**	8	51	8	58
**2**	40	79	40	118
**4**	13	70	13	65
**6**	6	48	6	25
**8**	0	18	0	0
**10**	0	0	0	0
**Mean (SD)**	2.5 (1.57)	3.3 (2.4)	2.5 (1.57)	2.4 (1.78)

Table 5. Distribution of 'need' and 'need non-Internet' scores amongst 333 respondents (11 missing values).

Internet Use

Nearly four out of five (78.8% (271/342)) had personally used the Internet in the last 3 months compared to 77% from national ONS sample in 2010. Table 6 shows the frequency of Internet use amongst those that had used the

Internet; 62% of the sample used the Internet at least once a day. Use of the Internet in the last three months was, as expected, strongly related to age. The PERQ sample (2012; sample=945, method=self-completed questionnaire, response rate 36%) was similar to ONS (2010; sample=1800, method=interview, response rate 59%) results for 2010 for different age groups (Table 7). There was no difference by gender in Internet use.

Frequency of use	Number (%)
Less than once a week	19 (7)
At least once a week	42 (15)
At least once a day	115 (42)
Many times a day	95 (35)
Total	271

Table 6.Frequency of Internet use amongst the 271 that had used the Internet in the last 3 months.

.

Age group	ONS 2010<br>% used Internet last 3 months	PERQ 2012<br>% (N) used Internet last 3 months
16-24	99%	100% (11)
25-44	96%	97% (85)
45-54	89%	89% (42)
55-64	78%	81% (52)
65+	40%	56% (58)

Table 7. Comparison of PERQ with ONS for age group ONS (2010; sample=1800, method=interview, response rate 59%)

Use of the Internet showed a gradient of use according to Zoopla estimated property price (χ^2=10.1; 3df; p=0.02), with 90% of those in the top quartile compared to 74% of those in the lowest quartile of house prices (Table 8). Although nearly half said they used it for health this was mainly to find information using a search engine. Few used discussion forums, Twitter, or Skype for health purposes (Table 9). (I describe later some inconsistencies between answers to B3 (as shown in Table 9) and section D of PERQ.

Zoopla estimated property value (grouped in quartiles)	Used Internet in last 3 months		Total
	Used	Not used	
<£101885	63(74.1%)	22 (25.9%)	85
£101885-145523	63 (70.8%)	26 (29.2%)	89
£145523-220113	71 (82.6%)	15 (17.4%)	86
>£220113	74 (90.2%)	8 (9.8%)	82
Total	271 (79.2%)	71 (20.8%)	342

Table 8. Use of the Internet by the Zoopla estimated property value of respondents

What did they use Internet for?	Any purpose<br>N (%)	For Health<br>N (%)
To find information (eg Google)	255 (94)	125 (46)
Email	257 (95)	14 (5)
Internet Telephone (eg Skype)	64 (24)	1 (0)
Discussion Forum	34 (13)	10 (4)
Twitter	22 (8)	1 (0)
Social network (eg Facebook, Linked In)	137 (51)	0 (0)
Watching videos (eg You Tube)	114 (42)	9 (3)
Virtual Worlds (eg Second life)	4 (1)	1 (0)

Table 9. What 271 people who had used Internet in last 3 months used the Internet for (Question B3)

Most people that used the Internet in the last three months did so at home (262/271) (Table 10). This was used to calculate a 'ubiquity score' (possible range 1-23) for those people who had used the Internet in the last three months (Table 11). Items were weighted, so home and mobile had weight 8, work had weight 4, and public or 'other' access each had weight 1. Anyone with score 8 or more had used the Internet either at home or on mobile access. Only 6/271 accessed the Internet only at work or in some other place.

Where	N (%)
Home	262 (97%)
Work	94 (35%)
Mobile	93 (34%)
Community	4 (1%)
Paid for	3 (1%)
Other	3 (1%)
Total	271

Table 10. Where people had used the Internet

Ubiquity score	Meaning	Number (%)
1-4	Only at work or some other place.<br>Did not access Internet at home or on mobile.	6 (2%)
8-9	Home or mobile, not work, but perhaps some other place	126 (46%)
12	Home or mobile and work	49 (18%)
16-17	Home and mobile, not work, but perhaps some other place	49 (18%)
20-23	Home, mobile, work, and perhaps some other place	41 (15%)

Table 11. Distribution of 'ubiquity' score: range of places people used the Internet (1 missing value).

Provision
Provision score comprises two main parts both based upon factual questions, Internet Provision (provisioninternet) and Internet Health Provision, (provisionhlth). The latter also comprises two parts: GP website, and Internet information or communication. These elements have been weighted: provisioninternet (4 points) and provisionhlth (5 points), comprising GP provision (3.5 points), condition specific information and support (1.5 point).

Internet Provision
Non-Internet-Users: 30 out of the 71 non-Internet-users had an Internet connected computer at home (provision score =1.5).Thirty nine did not have an Internet connected computer, but 10/39 knew that some of the neighbours had Internet access (provision score =1), 3 thought that the neighbours did not have access, and 28 did not know or did not answer (provision score=0).

Internet-Users: 249 people had used the Internet at home (B4) but 3 of these said (D1) they had no home Internet connection and 3 did not know. Two out of 3 who had accessed the Internet at home but had no home connection had used a mobile device, so it is possible that the wording of B4 needs to be changed slightly. Three people who had accessed the Internet at home but did not know if they had an Internet connection may have not known about the 'speed' of their home Internet, so question D1 may need to be changed slightly.

Of the 243 who had used the Internet at home, 211 (87%) thought their home Internet connection was fast enough and 13% (33) that it was not. Of a total of 36 people with no Internet connection or a connection that was too slow, 14 said it was because they would need to pay more, 12 thought their local server was congested and unreliable or their provider did not offer a faster connection, 1 lived in a rural area with only slow connections, and 9 did not know. Eighteen of those who had used the Internet had relatively low 'provision scores' (<1.5/3.5 max) including those who used the Internet only in places other than the home.

Internet Health Provision

Non-Internet-users were scored zero on Internet Health Provision. Of the 271 who had used the Internet 89 (33%) had looked at their GPs website, 51 (19%) thought their GP had one but had not seen it, 7 thought their GP did not have a website and the largest group (122, 45%) did not know. Of the 89 who had looked at their GPs website, 64 knew that you could order repeat prescriptions online, 6 said their GP did not offer this service, and 14 did not know. Only one person knew that they could see their record online, 20 knew that they could not, but 66 did not know.

Half (143, 54% (6 missing)) of those who had used the Internet (and answered this question (D6)) had tried to find information on health topics, all but nine of these having found what they wanted. The nine who had not found what they were looking for had been searching for information on BABY ADVICE, CONTRACEPTION METHODS, DENTAL INFO, HEMOCHROMATOSIS, LONG TERM USE OF STATINS, LOWERING CHOLESTRAL, MARINA COIL REMOVAL, and SKIN INFECTION. However, only 27 (11%) people had tried to contact an organisation or forum or other people online connected with health, of which 4 had not been able to contact who they wanted; 2 did not say who they tried to contact, one failed to contact a Breast Screening service and another a dentist.

Internet Health Provision score

Everyone has a GP, so one way of assessing the level of e-health provision is whether or not their GP provides a website and what facilities that website offers. As described earlier in this report, provision of information and support may vary by condition (e.g. lots of resources for breast cancer Vs fewer for stroke). However, not everyone has a need for health information or support and so may never have had reason to look for their GP website or for health information or support. It may also be that the reason they have not looked on the web is that they have not had the need or that their personal skills are not great. Provisionhlth has to be deduced from their answers to what they know about their GPs website and whether or not they have searched for and found information. We therefore used information about 'need' in allocating scores to provisionhlth.

We can only be sure that provision is not good, if they give clear negatives, ie

- they do NOT have a GP website (score 0/3.5)
- they looked for something on the web and it was not there (the latter may indicate lack of skill, but we can also argue that if a site is well hidden that is the responsibility of the information provider) (score 0/0.75)
- they tried to contact someone and were unable to do so (score 0/0.75)

If they do not know about their GP it is either because they have no need and so have not looked, or their personal skills are not great. However, to keep things relatively simple we do not add to 'Personal' from questions D3-D7, only to 'Provision'

GP provision is divided into 1 for a website, 1 for repeat prescribing, and 1.5 access to medical record

D3=1 implies good provision (and good personal skills) (+1/1)
D3=2 if need is positive – less good provision (or lesser personal skills) or if need is zero - nothing) (if need is positive (+0.5/1; if need is zero 0.75/1)
D3=3 implies poor provision (0/1)
D3=4 if need is zero implies nothing (+0.5/1) we take midpoint), if need is positive implies poor provision (or lesser personal skills) (+0.25/1)

D4=1 implies good provision (and good personal skills) (+1/1)
D4=2 implies poor provision (0/1)
D4=3 if need is zero implies nothing (we take midpoint)(+0.5/1), if need is positive implies poor provision (or lesser personal skills) (+0.25/1)

D5=1 implies good provision (and good personal skills) (+1.5/1.5)
D5=2 implies poor provision (0/1.5)
D5=3 if need is zero implies nothing (we take mid (ish) point) (+0.50/1.5), if need is positive implies poor provision (or lesser personal skills) (+0.25/1.5)

Condition specific information and support

D6=1 if need is positive implies poorer personal skills, if need is zero implies nothing (in both cases no action) (+0.25/0.75) (likely to be there, we just don't know)
D6=2 implies good provision (+0.75/0.75)
D6=3 implies poor provision (or poor skills) (0/0.75)

D7=1 if need is positive implies poorer personal skills, if need is zero implies nothing (in both cases no action) (+0.25/0.75)
D7=2 implies good provision (+0.75/0.75)
D7=3 implies poor provision (or poor skills) (0/0.75)

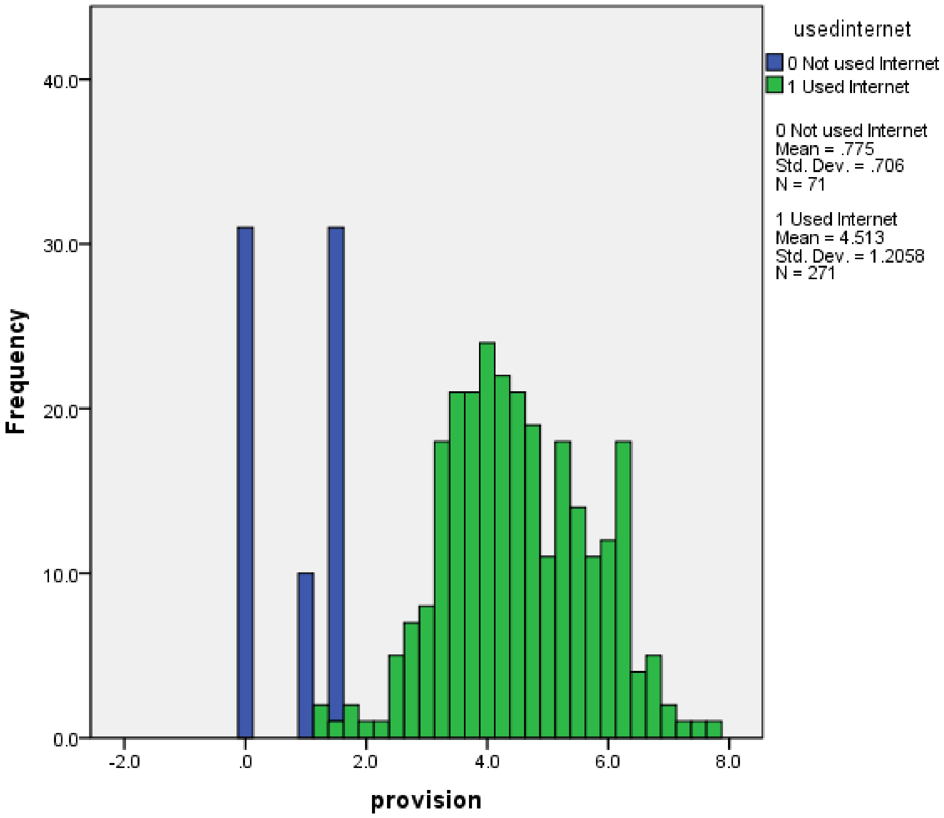

Figure 3. Distribution of provision scores, showing Internet and non Internet users

Face validity and possible improvements in provision scores
Figure 3 shows the distribution of provision scores comparing Internet-users and non-Internet-users. Extreme scores in provision were checked to see if they had face validity.

- (id 160) 37 year old F, seen doctor, read book/magazine. Uses Internet at least once a day. Googled for health, emailed, social networks, videos. Has a computer at home, work and mobile. Has looked at her GPs website, knows that it can be used for repeat prescriptions, does not know about medical record access, has searched for and found health information and used Internet to contact organisation for health. (Provision= 3.5+3.8=7.3).
- (id184) 50 year old F, seen doctor. Used Internet every now and then (Googling and email but not for health). Used it at a community centre. Has not got a home computer, does not know if the GP has a website, never tried looking for health information. (Provision =0+1.3=1.3). (Someone who has used the Internet, even though they may not have tried searching for health information, has in principle access to Internet health resources, so not score zero).
- (id155) 51 year old F, seen doctor. Not used Internet but has an Internet connected computer at home (no-one has used computer to get health information for her, but she would have a go at using the Internet if someone helped her). (Provision = 0+0=0).

Personal
The personal score assesses:

- A moderated confidence score for those who have accessed the Internet
- A willingness to try using the Internet for those who have not

So the personal score is based questions that ask for ratings, but asks different questions depending on whether the person is an Internet user or not, and uses one set of ratings to moderate another.

Twelve people (3.5%) said they had disabilities that made using computers difficult, 6 were people who had not used the Internet in the last 3 months (arthritis, eye problems, 4 not stated) and 6 had used the Internet (hearing impaired, learning difficulty, ME/dyslexia, neuropathic arm pain, Parkinsons, photosensitivity).

The personal score for non-Internet-users was constructed from three questions about their willingness to 'have a go' (C6, C8, C9). Eight of 71 non users did not answer the question, of the 63,

- 28 (44%) would possibly, probably or definitely have a go at using the Internet if they had help (C6)
- 29 (44%) would possibly, probably, or definitely use a home Internet connection if they had help and it was cheap (C8)
- 29 (44%) would use the Internet for health at some other place (Table 12).

Place	Number
A place of work	2
A public library	12
A community centre, or Age UK centre	5
A place of worship	1
Your doctor's practice	4
Your local hospital	0
The house of a family member	1
A friend's house	7
Other?	1
**NONE of these**	3

Table 12. Places where non Internet users might be prepared to use the Internet for health .

For 271 Internet users, PERQ asks people to rate their skills on four tasks (E2) (Table 13). This serves two purposes: (i) to 'ground' their confidence rating in the reality of their ability, and (ii) to provide a consistency check on their confidence rating. We derived a 'skills' score (0-12) from question E2 (adding 0 not answered, 1 no, 2 maybe, and 3 yes for each of the four questions). The derived skills score (0-12) was correlated with the E3 self-rating (Pearson Correlation=0.58, $p<0.001$), nevertheless there were some outliers (shaded cells), i.e. four people who rated their skills low but confidence high and 13 people who rated their skills high but confidence low (Table 14).

Internet Task	No	Maybe	Yes	Missing
Book tickets for film and save copy into folder	25	32	200	14
Search Google and open first 3 sites	12	16	231	12
Use Google to find documents for new passport	17	32	209	13
Compare cost and convenience	19	42	199	11

Table 13. Self-rating of skills amongst 271 Internet-users

Skills score from E2	Confidence rating from E3				Total
	0-2	3-5	6-7	8-9	
**0-3**	3	3	0	1	7
**4-6**	12	5	0	1	18
**7-9**	12	12	4	2	30
**10-12**	13	37	70	91	211
**Total**	40	57	74	95	266

Table 14. Comparison of self-rated skills score with self-rated Internet confidence score

Given that questions E2 (skills) and E3 (confidence) were in place of the e-health literacy scale, and given our findings from an earlier round of piloting these two questions seem to work quite well, but so that there is more consistency a ‘moderated’ confidence scale of E3*E2/12 has been calculated.

I wanted to check whether people’s (moderated) confidence in their use of the Internet for health was associated with their frequency of use, range of uses, and ubiquity of use. From this model I have assumed that ‘personal’ is one of the contributors to how much use someone makes of the Internet and so should be measured separately. Nevertheless they should be associated. To explore this I compared the ‘moderated confidence’ scores with range, frequency, and ubiquity of use (Table 15).

As would be expected there was a strong association between frequency of use and moderated confidence ((χ^2=81, 12df, p<0.001). Nevertheless there were still some ‘outliers’, for example one person (shaded cell) who was very confident despite using the Internet less than once a week. The four people who used the Internet many times a day but had low confidence is more ‘believable’ as they may use it for just limited purposes. Similarly, as would be expected there was a strong association between range of use and moderated confidence ((χ^2=61.5, 12df, p<0.001) but again there were still some ‘outliers’, e.g. 8 people who only ever used the Internet but whose moderated short confidence score was 5. The range of uses of the Internet for health was less and the association slightly less (p=0.002).

Finally, the moderated confidence score had a strong association with ‘ubiquity’, i.e. the places where people accessed the Internet ((χ^2=81, 16df, p<0.001). Some might argue that range of health uses should be the outcome measure of any intervention. However, this will be dependent on someone’s need for health information. As we can see above they are associated but the moderated confidence score seems a ‘cleaner concept’. So PERQ will report both but not incorporate range of health uses into e-health readiness, but perhaps use it as a secondary outcome measure.

**SM CS**	**Frequency of Internet Access**				**Range of use (any purpose)**				**Range of use (health)**			**Places where Internet used**					**Tot**
	1	2	3	4	1	2	3	4	0	1	2	1	2	3	4	5	
1	6	2	4	4	15	1	0	0	14	2	0	2	12	1	1	0	16
2	11	13	24	9	34	18	5	0	40	17	0	3	39	10	5	0	57
3	1	17	32	13	20	34	9	0	30	33	0	1	40	7	12	3	63
4	0	7	37	40	25	35	22	2	36	48	0	0	25	17	18	24	84
5	**1**	3	18	29	**8**	23	16	4	22	28	1	0	10	14	13	14	51
Tot	19	42	115	95	102	111	52	6	142	128	1	6	126	49	49	41	271

Table 15. Short moderated confidence score (SMCS) vs Frequency of access, Range of uses of the Internet for any purpose, Range of uses of the Internet for health, Places where the Internet was used.
Key:
Frequency (1: less than once a week, 2: at least once a week, 3: at least once a day, 4: many times a day),
Range of uses of the Internet for any purpose (count),
Range of uses of the Internet for health (count)
Places (1: only at work or elsewhere, 2: Home or mobile but not at work, 3: home or mobile and work, 4: home and mobile, not work, 5: home, mobile, and work).

Face validity and possible improvements in personal scores
Figure 5 shows the distribution of personal scores comparing Internet users and non-users. Despite ‘grounding’ the estimates of self-efficacy by asking (just before) about skills, there were still a large minority (51 (15.3%)) of the sample with scores with the maximum of the range (9), being able to do all four Internet tasks and being totally confident in their use of the Internet. (This means that these people would not be able to increase their personal score during the course of a study (ceiling effect)). This might suggest that some ‘harder’ tasks are included in E2, and to focus the questionnaire better on e-health this should perhaps include some health focused questions.

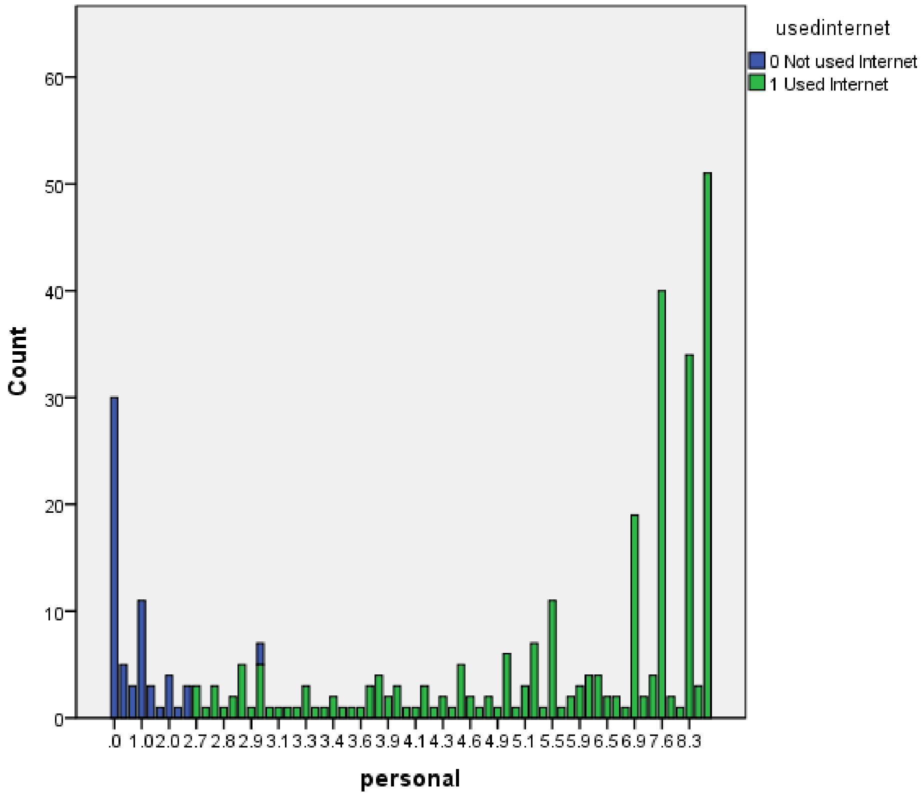

Figure 4. Distribution of personal scores, showing Internet and non Internet users.

Support
Although the opinions of the respondents are embedded in their answers, the support score is largely based on factual questions. For non-Internet-users the support score is derived from C5 and C7 (maximum of 6), and for Internet users it is derived from section F. Twenty two out of 271 Internet users did not complete section F, half of these (10/22) said (H1) that they had no barriers to Internet use, and were confident in using the Internet for health (E3). This has been 'dealt with' in the way 'support' and 'personal' are combined into the e-health readiness score (see below). For Internet-users each question can add to the support score as follows: F1 (+2), F2 (1.5), F2XX (+1), F4 (+1.5), F5 (+1.5), F6 (+1). Figure 5 shows the distribution of support scores for Internet users and non-Internet-users.

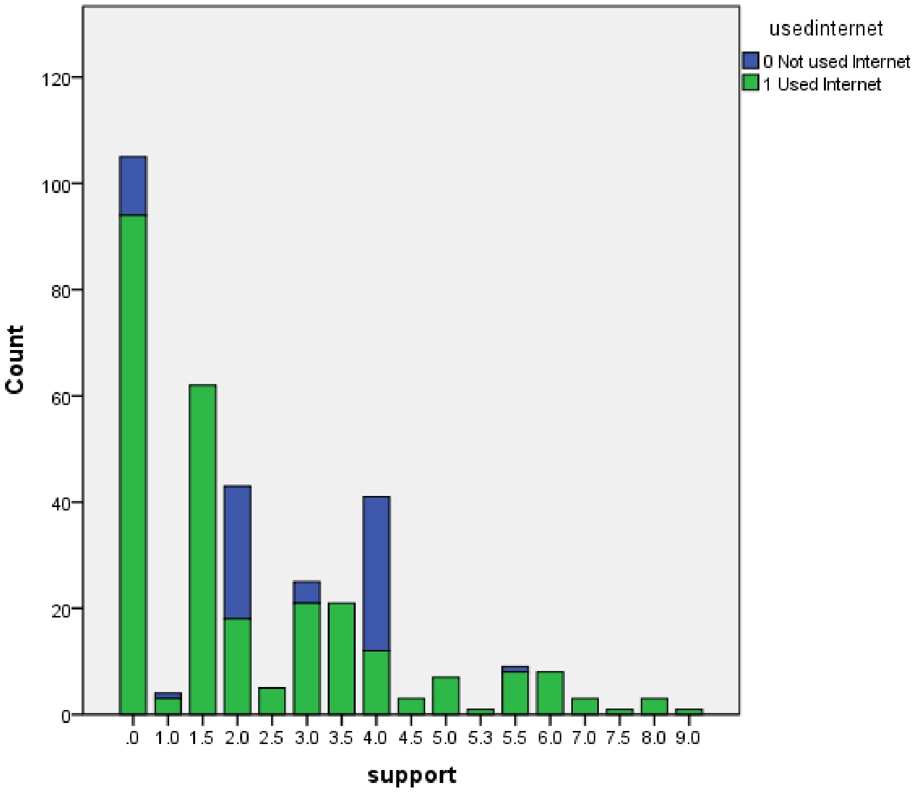

Figure 5. Distribution of support scores, showing Internet and non Internet users

Among Internet-users, only 58 (21%) had been given information by a health professional to help them use the Internet. Just under half (117, 43%) knew where they could find help locally in using the Internet and many of these (78) cited their local library. Other sources of help listed included University or college (13), family (12), friends and neighbours (5), Age UK (4), NHS (3), local computer firm (3), colleagues, community centre, and Internet café. However, only 20 (7%) people had made use of such help. A quarter (68/243 (question F3) said there had been times when help would have been useful, and of these, 50 had someone they could ask, of which 47/50 could ask about health.

Nearly three-quarters (49, 73% of those who answered) of non-Internet-users had had someone use the Internet for them. Eighteen people had not used the Internet in the last three months and had never had someone use the Internet for them. Forty people (65%) had someone that could help if they wanted to have a go at using the Internet. Five had help but it would not be easy to ask as they lived too far away.

Economic
The economic subscale was constructed slightly differently to the other three relying on comparison of perceptions of the cost of using the Internet compared to other health activities such as visiting their GP or local hospital. As such it was probably the sub-scale most like a ‘traditional’ rating scale. Both Internet-users and non-Internet-users answered the same questions.

There were significant differences on the two Internet questions and on the cost of visiting hospital between Internet users and non-users. For the two internet questions this was dominated by the ‘don’t knows’ amongst non-users; 45% of non-users did not know about the cost of home access and 57% about the cost of mobile access compared to 5% and 33% of Internet users. There was no difference between users and non-users in perceptions of cost for local library or GP. Most (60%) did not think it cost much to get to a public library to use the Internet, but a large minority (95, 29%) did not know. The vast majority (94%) agreed that visiting their GP cost nothing or very little. Non-users were more likely to think that visiting their nearest hospital cost nothing or little (82% Vs 69%; χ^2=15.7; 4df; p=0.003); this may be because more had bus passes. This may be an important reason why Internet appears relatively more expensive to older non-users. Figure 6 shows that the non-Internet-users were likely to have lower economic scores overall (χ^2=39; 8df; p<0.001).

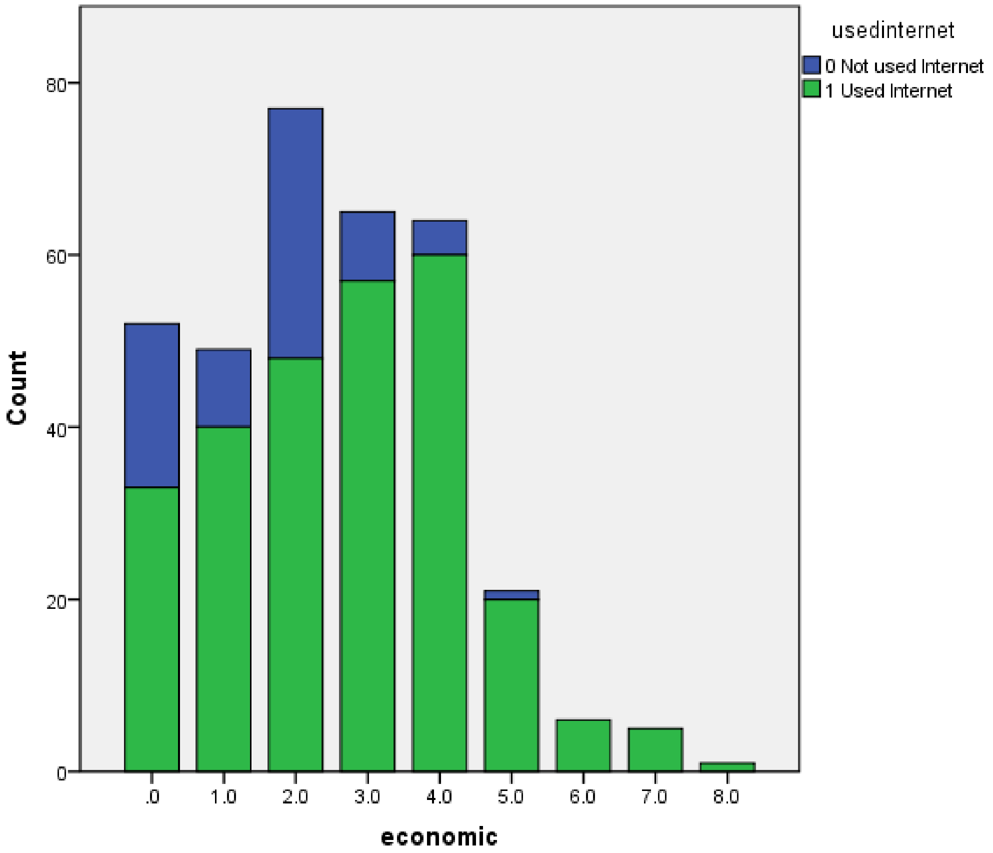

Figure 6. Distribution of economic scores, showing Internet users and non-users (Higher score means participants think Internet access is not expensive).

Overall view on using Internet for health
Question H1 sought to identify the most important issue (if any) in using or not using the Internet for health. The original intention was to use this question to weight the sub-scale scores in their combination to produce an overall readiness score. This idea was abandoned when it was realised that there was a close relationship between the support and personal subscales and an alternative combination method was developed. However, H1 remains a useful consistency check on the subscale scores. Table 16 shows that most Internet-users (185, 70%) thought they had no real barriers to using the Internet for health. Among non-Internet-users 61% said they had no interest in using the Internet.

Overall View	Non Internet User		Internet User	Total
	Home access	No home access		
1. No need for health information	1	3	34	38
2. No interest in using the Internet	11	29	12	52
3. Would use the Internet more for health if could get a good Internet connection	0	2	3	5
4. Don’t understand the Internet that much	9	4	17	30
5. Would use the Internet more for health if could get someone to help	0	0	10	10
6. Would use the Internet more for health if money were no object	1	2	2	5
7. Uses or would use the Internet for health and have no real barriers to that use	4	0	185	189
Total	26	40	263	329

Table 16. Overall view (15 missing values).

Further breakdown of the groups in Table 16 show the range of different situations and attitudes. Of the 52 with ‘no interest in using the Internet’, 12 had used it in the last three months and 40 had not. Of these 40, 11 had home Internet access but had not used it personally (but most had someone else use it for them). Twenty nine (of 52) did not have home Internet access. Relatively few chose connectivity, economic reasons, or need for support as the main barrier to Internet use for health.

We would expect that the constructed sub-scales would show an association with responses to question H1. To see if this was the case I compared the constructed variables against answers to question H1 (Table 17). All but H1=3 (‘would use Internet more if could get a better connection’) and H1=5 (‘would use Internet more if could get someone to help’) showed significant differences on the expected variable. As Table 16 had shown, there were few that chose these two responses.

(H1) Overall views about using Internet for health	‘Nearest’ variable	Mean score those who chose this item versus rest	T-test
1.No need for health information	NEED	1.8 Vs 3.3	(t=3.8, p<0.001)
2.No interest in using Internet	PERSONAL	1.1 Vs 6.2	(t=14.2, p<0.001)
3.Connection	PROVISION	No difference	
4.Don’t understand	PERSONAL	2.7 Vs 5.7	(t=5.2, p<0.001)
5.Someone to help	SUPPORT	No difference	
6.Money	ECONOMIC	0.8 Vs 2.5	(t=2.3, p=0.025)
7.No barriers	PERSONAL	7.2 Vs 3.2	(t=-16.8, p<0.001)
	ECONOMIC	3.0 Vs 1.9	(t=-6.3, p<0.001)
	PROVISION	4.0 Vs 1.9	(t=-13.7, p<0.001)
	SUPPORT	3.1 Vs 2.3	(t=-4.6, p<0.001)
	READINESS	4.4 Vs 2.3	(t=-16.1, p<0.001)

Table 17. Independent sample t-tests to assess correspondence between overall view and constructed scores.

Face validity of ‘short’ scores

	Provision		Personal		Support		Economic	
**Score**	Not used Internet	Used Internet	Not used Internet	Used Internet	Not used Internet	Used Internet	Not used Internet	Used Internet
0	31	0	38	0	11	94	19	33
1	40	19	23	16	26	88	38	88
2	0	154	2	57	33	57	12	117
3	0	93	0	63	1	24	1	26
4	0	5	0	84	0	7	0	6
5	0	0	0	51	0	1	0	0
Total	71	271	63	271	71	271	70	270
Mean (SD)	0.56 (0.50)	2.31 (0.63)	0.43 (0.56)	3.34 (1.18)	1.34 (0.75)	1.13 (1.09)	0.93 (0.71)	1.57 (0.90)

Table 18. Distribution of ‘short scores’ for Internet users and non-users.

Table 19 gives examples for the four dimensions of respondents who had not used the Internet that had higher scores or the same scores as Internet-users, as shown by the shaded cells in Table 18. These are particularly of concern to see if the merger of scores from Internet and non-Internet-users ‘makes sense’.

PROVISION
(ID=8) Female aged 86, had not used Internet, no home connection, neighbours have connection (provision short score = 1).<br>(ID=184) Female aged 50, had used Internet at a community centre, D1 and D2 not answered, D3 don't know, D4 not answered, D5 don't know, D6 never tried, D7 never tried. (provision short score = 1).
**PERSONAL**
(ID=212) Female aged 45, had not used Internet last 3 months, used to use it fairly often but not recently, does not have a home computer, if someone was able to help she would have a go at using it, if it was easy and cheap she would have a home connection, she would use computers elsewhere (family or friend), (personal short score=2)<br>(ID=79) Female aged 82, had used Internet, uses it every now and then, has a home computer, could not do any of the four skills tasks, confidence=1 (personal short score=1)
**SUPPORT**
(ID= 1) Female aged 62, had not used Internet, someone has used Internet for her, and she would be able to ask someone to help have a go on Internet easily (support short score =2)<br>(ID=10) Female aged 90, uses it at least once a day, had not been given help by health professional, did not answer F2, there were not times when help would have been useful so did not answer question F4-6 (support short score =1)
**ECONOMIC**
(ID=130) Male aged 64, had never used Internet, but house has an Internet connected computer. Cost of home internet was not a concern, did not know about cost of mobiles, getting to a library to use a computer did not cost much, thought getting to hospital was expensive (economic short score = 3)<br>(ID=6) Male aged 62, used the Internet many times a day, had home Intenet. Cost of home internet was not a concern, thought mobile access was expensive, did not know about cost of getting to library, did not think getting to GP or hospital was expensive (economic short score = 2).

Table 19. Examples of 'pairs' where non Internet user has higher or same score as Internet user.

Combining provision, personal, support, and economic subscales into an overall 'readiness' score and the face validity of that score

The initial intention was to create an overall readiness score based on the sum of the four subscales, that is (provision + personal + support + economic)/5. However, exploration of the data and further consideration of the importance of 'support' suggested that support was much more important for non-Internet-users or those with a limited range of uses. Those who were already competent users of the Internet for health needed little support and therefore scored low on support. This reduced their overall readiness score and was misleading. Support was therefore added to 'readiness' in proportion to that person's personal score. By trial and error and looking at whether the impact on overall readiness made 'sense', the term 3*support/(personal+support) was added as the contribution to readiness. The modified support score can range from 0 to 3 and the sum of personal and modified support can range from 0 to 10.5. Table 21 shows the contributions to readiness of different combinations of personal and modified support. The economic score also seems less important in being 'e-health ready' than personal and provision scores so we have used the 'short score' (range 0-5) as the contribution to readiness. So

Readiness= Provision (0-9) +
Personal + modified Support (0-10.5) +
Short economic (0-5)
and then scaled to be in the range 0-9.

Support	Personal	Personal + support	Modified Support	Personal + modified support
0	0	0	0	0
4	0	4	3	7
0	4	4	0	4
4	4	8	1.50	5.5
0	9	9	0	9
4	9	13	0.92	9.92
9	9	18	1.50	10.5

Table 20. Contribution to the overall readiness scale of the combined personal+modified support term

The maximum possible range of the e-health readiness scores is 0 to 9. In this sample scores ranged from 0-7 with mean (SD) 4.1 (1.79 (Figure 7). People who had not used the Internet had, as expected, lower scores than those who had used the Internet.

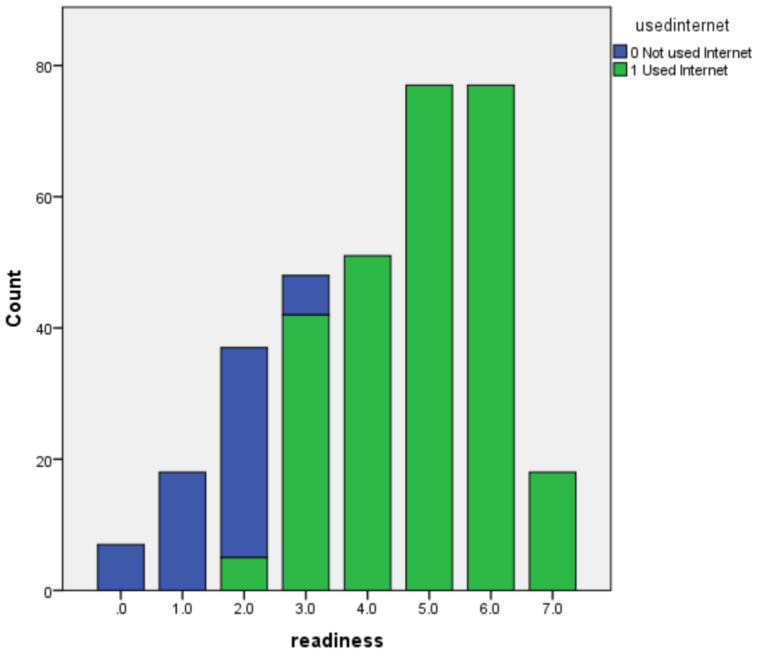

Figure 7. Distribution of e-health readiness scores on possible scale 0-9 showing internet-users and non-internet-users

Repeatability and change over time

Seventeen of the 20 people who completed the second stage pilot in January 2012 were asked and completed a PERQ4 in September 2012. Of these, four non Internet users were excluded as section C had changed too much between the earlier and later version of PERQ to be used. For the remaining 13 we used the January data to ‘complete’ the September version of the questionnaire to allow a comparison and some assessment of ‘repeatability’ and change over time. Each pair of questionnaires was examined for changes to answers and the impact on the scoring system to see if ‘it made sense’ and if the scoring system was appropriate. This check resulted in some changes to the scoring system. With the final scoring system Figures 9 to 13 show reasonable consistency in scores between January and September 2012. One person (ID 9) was an outlier on ‘provision’ (with provision score 8 in September), on ‘personal’, and on ‘economic’. On overall ‘readiness’ she improved from 4 to 8; the other 12 all had the same readiness scores in January and September 2012. She had acquired a smart phone or tablet between January and September 2012. In January, she did not know if her GP had a website but by September had looked it and knew that she could use it for repeat prescriptions and to see her own medical record online. She had a much more optimistic view on economics in September. A different person (ID=18) was an outlier on ‘support’ with score 4.5 in September (ID =18). This 77 year old woman had made use of help (F2) in accessing the Internet by September.

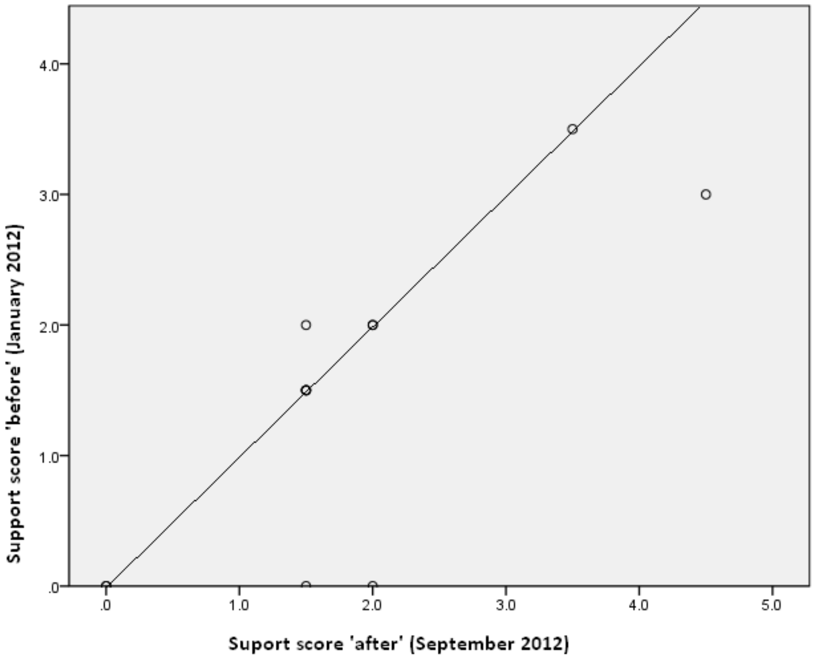

Figure 8. Support scores, before and after for 13 people who completed PERQ in January and September 2012

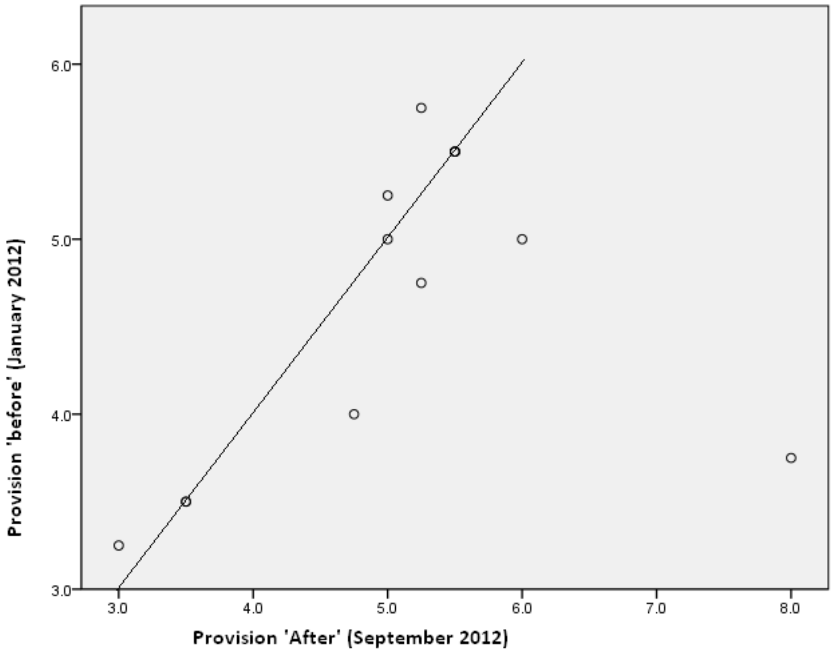

Figure 9. Provision scores, before and after for 13 people who completed PERQ in January and September 2012

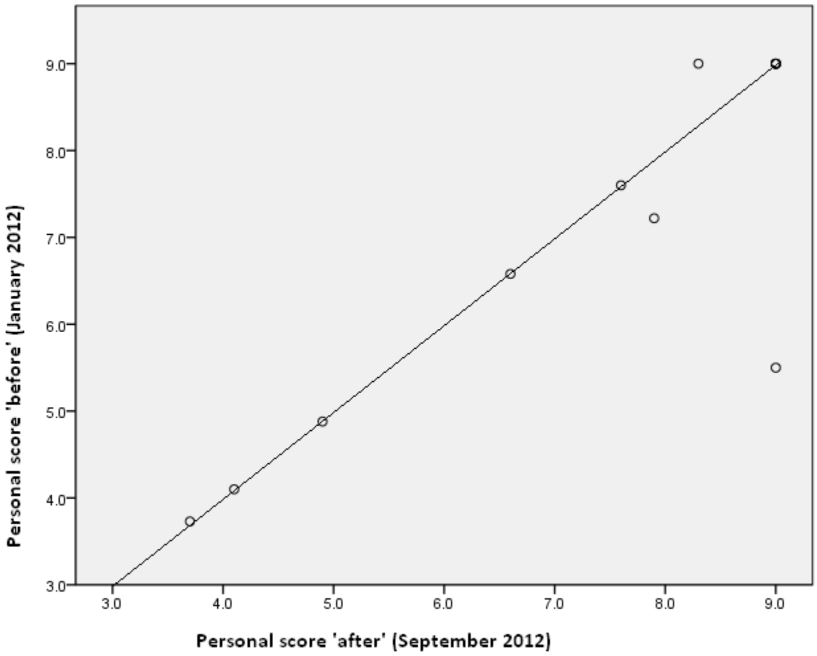

Figure 10. Personal scores, before and after for 13 people who completed PERQ in January and September 2012

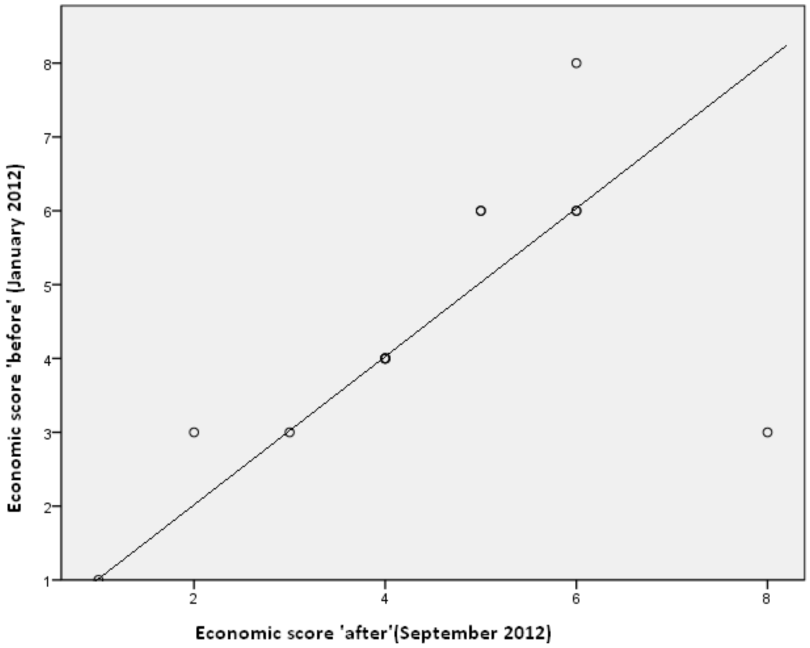

Figure 11. Economic scores, before and after for 13 people who completed PERQ in January and September 2012

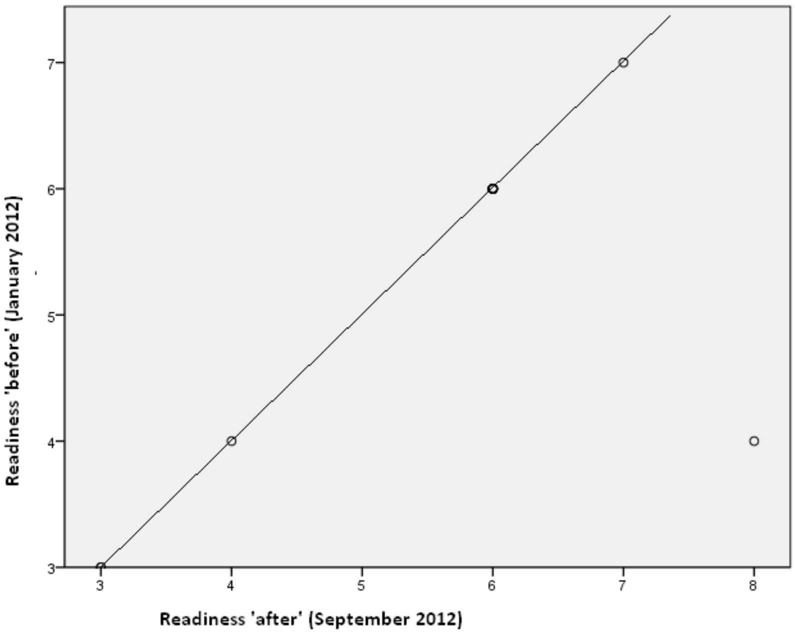

Figure 12. Readiness scores, before and after for 13 people who completed PERQ in January and September 2012

3. QUESTIONNAIRES

PERQ1
PERQ2
PERQ3
PERQ4 Version used in baseline survey

The questionnaires have been ‘squashed’ slightly to fit whole pages in this report with the addition of page numbers. In use, the questionnaires did not have page numbers. Labels (PERQ1, 2 etc) have also been added to the title to help identify the different versions. In use, these labels were not present.

Using the Internet for health care in Plymouth (PERQ1)

We want to hear from everybody, not just those who have used the Internet or are interested in using it.

My name is Professor Ray Jones and I am hoping that you will be able to help with this survey being carried out in Plymouth. The survey is about using the Internet for health care, but we want to hear from everybody regardless of whether or not you use it.

Completing the attached questionnaire should take about 10 minutes. Let me explain why we are doing this survey and why a response from your household is important.

Why are we doing this study?
A lot of people have found the Internet useful for their health but not everyone can access or wants to use it. Moreover, the NHS could improve services and save money (to be used in other parts of the NHS) by making better use of the Internet. Of course, not everyone wants to do things that way but it is important that everyone can have the opportunity. So our concern is about inequalities in being able to use the Internet for health.

We are concerned that not everyone has the opportunity to use the Internet for healthcare. At the moment we do not have a consistent way of assessing different people's opportunity and desire to use the Internet for healthcare. We need to make sure that any questionnaire 'works' for those who are not interested in the Internet as well as those who use it a lot. Therefore, to do that, we are developing and testing the questionnaire in this survey of Plymouth.

Why should you complete and return this questionnaire?
We are hoping that one person in your household will complete and return the questionnaire in the reply paid envelope. There are instructions on the first page to find out who should complete the rest of the questionnaire. It will help us with our research and that research will eventually lead to a better understanding of inequalities in using the Internet for health.

As an extra incentive:

- We will have a prize draw for each district in the study. The winner will receive an Amazon voucher for £20. You have a good chance (about 1 in 60) of winning.
- You can receive a free printed copy of a guide to the Internet. If you are interested please tick the appropriate box on the last page of the questionnaire.

What will we do with your completed questionnaire?
We will be analysing the data to make sure that the questionnaire and survey methods 'work'. If you have any comments or suggestions about the survey please do let us know by writing in the space provided. The number on the questionnaire tells us which house it came from, to enable us to give the winner their Amazon voucher. Otherwise the questionnaire is anonymous. We will keep the questionnaires in a locked cabinet until we have finished the survey. They will then be destroyed.

Thank you very much for your help. If you have any questions or comments before completing the questionnaire please contact me: Professor Ray Jones, Faculty of Health, Education and Society, Plymouth University, 3 Portland Villas, Plymouth. (ray.jones@plymouth.ac.uk)

Using the Internet for health care in Plymouth

We want to hear from everybody, not just those who have used the Internet or are interested in using it.

Your household and who should complete the questionnaire

Please can you write in the box below the ages of everyone who is living in your household today, putting the ages of males in the left hand box and females in the right hand box? * Then circle the person who is aged 16 or more and who has the next birthday. That is the person who we would like to complete the questionnaire. It does not matter if that person has never used the Internet and has no interest in it.

Example: if there is 'mum', 'granny,' 'dad', and 'son' and it is 'granny' that has the next birthday the box might look like.

MALES (example)	FEMALES (example)

Please complete this section below.
The person circled should complete the rest of the questionnaire.

MALES	FEMALES

If the person with the next birthday needs help in completing the questionnaire (e.g. if they have poor eyesight or are unwell) please tick this box [ ] (Completed by someone else on behalf of the person with the next birthday.)

(* The reason for asking about all people in the household is so that we can know how our sample of respondents compares to the 'demographics' of Plymouth's population. Also the composition of households may influence the ability to get help in using the Internet.)

Using the Internet for health care in Plymouth

We want to hear from everybody, not just those who have used the Internet or are interested in using it.

A. HEALTH INFORMATION AND SUPPORT

In the last three months have you ever (tick ✓all that apply):

Seen a doctor, nurse, or other health professional about your health [ ]
Asked a family member or friend something about your health [ ]
Phoned a helpline (e.g. NHS Direct, Samaritans, Diabetes UK) about your health [ ]
Read a book, or magazine to find something out about health [ ]
Used the Internet for something to do with your health [ ]
None of these [ ]

B. INTERNET USE

This section is about whether you have used the Internet, how often and where you use it.

B1. Have you personally used the Internet in the last 3 months? Yes [ ]   No [ ]
If yes, continue with **question B2 below**
If no, go to **question C1 on the next page**

B2. Typically how often do you use it?
Many times a day [ ]
At least once a day [ ]
At least once a week [ ]
Less than once a week - every now and then [ ]

B3. What have you used the Internet for? Tick boxes in the first column for all the ways you have used the Internet for any reason, and tick boxes in the second column if you have used the Internet in that way for something related to your health.

	Have used the Internet for…..	
	Any purpose	For something health related
Browsing web pages		
Email		
Instant messaging		
Internet telephony (eg Skype)		
Discussion forum		
Twitter		
Social network site (eg Facebook, Linked in)		
Watching videos		
Virtual World (e.g. Second Life)		

B4. Where and how have you accessed the Internet in the last 3 months? Tick all that apply.
Desktop / laptop computer at home [ ]
Desktop / laptop computer at work [ ]
Smart phone or mobile device such as iPAD [ ]
Desktop computer in a library or community centre [ ]
'Paid for' computer in an Internet café, shop, airport [ ]
Computer at someone else's home [ ]
Computer at GP's practice or hospital [ ]

C. FOR PEOPLE WHO HAVE NOT USED THE INTERNET IN THE LAST THREE MONTHS
This section asks about whether you would like to use the Internet, if maybe you would like to use it for health related things, given help.
If you HAVE used the Internet in the last three months, go to section D.

C1. Have you ever used the Internet? (Tick one of the following)
I used to use it fairly frequently but not recently [ ]
I have only ever used it a few times and not recently [ ]
I have never used it [ ]

C2. Does your home have an Internet connected computer? Yes [ ] No [ ]

C3. Has anyone ever used the Internet for you, e.g. to find out something for you, or to buy something for you, or to contact someone on your behalf by email? Yes [ ] No [ ]

C4. If someone was able to help you, and it was easy, and it was cheap, would you make use of a home Internet connection? No [ ] Possibly [ ] Probably [ ] Yes [ ]

C5. In this table, tick the left hand column if you go there in a typical month, tick the middle column if you would use an Internet connected computer there (assuming that it was free, easy to use, and there was help to use it) for any purpose, and tick the right hand column if you would use it there to get health information.

	Tick if……		
Places you might go to in a typical month	You go there	You would use the Internet there if given the opportunity	You would use the Internet there for health information
A place of work			
A public library			
A community centre, or Age UK centre			
A place of worship			
Your doctor's practice			
Your local hospital			
The house of a family member			
A friend's house			
Other?.......			

There is just one more page of questions that we would like you to complete.
Please go to section G on the last page.

D. Provision of Internet services.
Answer this section if you have used the Internet in the last three months.

D1. Does your home (tick ✓ one)……..
Have an Internet connection that is fast enough for you [ ]
Have an Internet connection that is slow for what you need [ ]
Has no Internet connection [ ]
Don't know [ ]

D2. Could your home currently have a 'fast enough (for you)' Internet connection?
(Tick ✓ one only)
It already does [ ]
Probably, if I paid for it [ ]
I don't think so (because of rurality, too much 'traffic', no provision etc) [ ]
Don't know [ ]

D3. Does your General Practitioner (family doctor) have a website?
Yes I have looked at it [ ]
Yes I think so but I have not seen it [ ]
No [ ]
Don't know [ ]

If your General Practitioner (GP) has a website:

D4. If you wanted, can you order a repeat prescription by email, or on your GP's website? Yes [ ] No [ ] Don't know [ ]

D5. If you wanted, can you see your own medical record online via your GP's website? Yes [ ] No [ ] Don't know [ ]

In the last three months, have you used the Internet trying to….

D6. Find information about health topics, services, treatments, advice etc.)?

Never tried [ ]
Tried, and found what I wanted most of the time [ ]
Tried, but not been able to find what I wanted [ ]
Can you say what topic?...

D7. Contact an organisation, or discussion forum, or other people, for some reasons connected with your health and been able to get what you wanted?

Never tried [ ]
Tried, and found what I wanted most of the time [ ]
Tried, but not been able to contact who I wanted [ ]
Can you say who/what organisation you were trying to contact?..........................

E. PERSONAL SKILLS AND CONFIDENCE IN USING THE INTERNET FOR HEALTH

This section is for people who have used the Internet in the last three months. It is about your own physical ability, knowledge, skills, interest, and emotional state in using the Internet.

E1. Do you have a permanent physical disability that makes using the Internet difficult?

No [ ]
Yes - makes use of the Internet very difficult [ ]
Yes - makes use of the Internet somewhat difficult [ ]
If Yes, please give details………………..

E2. Do you have, or have you had, a physical or mental condition that means makes using the Internet difficult just at the time when maybe it would be useful?

No [ ]
Yes – when I feel ill/bad, using the Internet is very difficult [ ]
Yes – when I feel ill/bad, using the Internet is somewhat difficult [ ]
If yes, please give details…………………….

E3. This question is a self-assessment of your general Internet skills, not necessarily concerned with health. In the following table, please read the 'task' and then tick one box to show if you think you could do that task.

I think I could…..	No	Probably	Yes
Book tickets for a film online and save a copy of the booking into a folder on your computer			
Search on Google with keyword 'Cancer', and open the first three sites found, to compare what they say			
Use a search engine (e.g. Google or Bing) to find out what type of documents you need to apply for a new passport if yours was lost			
Compare the cost and convenience of a holiday using a 'package holiday' company to booking travel & accommodation separately			

E4. This question asks about your confidence in using the Internet for health. Please tick one box for each line.

	**Strongly disagree**	**Disagree**	**Not sure**	**Agree**	**Strongly Agree**
I know what health resources are available on the Internet					
I know where to find helpful health resources on the Internet					
I know how to find helpful health resources on the Internet					
I know how to use the Internet to answer my health questions					
I know how to use the health information I find on the Internet to help me					
I have the skills I need to evaluate the health resources I find on the Internet					
I can tell high-quality from low-quality health resources on the Internet					
I feel confident in using information from the Internet to make health decisions					

E5. In general how confident are you in using the Internet? Circle a number between 1 (not at all confident) and 10 (totally confident).

Not confident………………………………………………………………..Totally confident									
1	2	3	4	5	6	7	8	9	10

F. INTERPERSONAL SUPPORT IN USING THE INTERNET FOR HEALTH
This third section is about whether you have, or could have if you wanted it, support from somebody in using the Internet for health.

F1. Do you have a family member or friend who has, or could, help you to use the Internet (for any purpose)? (Tick one)
No [ ]
Yes, there is someone I can ask quite easily [ ]
Yes, but they are not, or would not be very easy to ask [ ] Why is that?..............................

F2. **If yes to question F1,** would you feel OK about asking them to help you use the Internet for **health** purposes (to find information or to communicate with someone)?
Yes [ ]
No [ ]   If no, why is that?..

F3. Has a doctor, nurse, or other health professional ever given you information (e.g. a web address) on how to use the Internet for your health? Yes [ ]   No [ ]

F4. Are you able to get help using the Internet for health from an organisation or society (e.g. from the local library, Age UK, local authority, NHS, or University run service)
[ ] No, not that I am aware of
[ ] Yes, from …………………………………………………………

If yes, have you ever made use of such help? Yes [ ]   No [ ]

F5. If you were to get support from family or friends in using the Internet for health would you have any concerns about disclosing information about your health to?
[ ] No, not really
[ ] Yes, please give details……………………………………………………………………

F6. If you were to get support from an unknown person online or by phone in using the Internet for health and you were anonymous to them would you have any concerns about disclosing information about your health to?
[ ] No, not really
[ ] Don’t know
[ ] Yes, please give details……………………………………………………………………

F7. If you were to get support from family or friends in using the Internet for health would you have any concerns about being dependent on that person for help?
[ ] No, not really
[ ] Yes, please give details……………………………………………………………………

G. ECONOMIC CONSIDERATIONS IN USING THE INTERNET FOR HEALTH

This section asks about the cost to you of using the Internet for health

[illegible] ease tick one box as your best guess even if you do not have the Internet).

£5	£10	£15	£20	£30	£40	£50	£60	£70	£80

G2. Please tick ✓ one box for each line to show whether you agree or disagree with each of the following statements, as they relate to you at the moment. They include statements about using the Internet but also about visiting your GP or hospital, for comparison.

	Strongly agree	Agree	Disagree	Strongly disagree	Don’t know, don’t care
The monthly cost of home Internet is a major concern					
Mobile Internet access on smart phones and iPADS is expensive					
Getting to a public library to use the Internet does not cost much					
It costs me nothing, or very little, to get to see my GP					
It costs me nothing, or very little, to visit my nearest hospital					

H. What are your thoughts about the Internet and health?

This last section is an ’open section’ for your comments. Do you have any thoughts about using the Internet for health? In particular, do you have any ideas on what could be done to help those who want access to the Internet for health, but do not have it either (a) because of lack of physical access, or (b) need to help or training, or (c) need for support from someone, or (d) because of the cost?

…………………………………………………………………………………………………..

…………………………………………………………………………………………………

…………………………………………………………………………………………………

Thank you very much for your help in completing the questionnaire. If you send the questionnaire back in the prepaid envelope you will be entered into the prize draw for an Amazon voucher. The following six guides are all available on the Internet if you have access, but if you would like us to send you <u>one</u> free printed copy, please tick the appropriate box. Please tick one box only.

From Age UK	From the BBC
www.ageuk.org.uk/work-and-learning/technology-and-internet/<br>[ ] Making the most of the Internet<br>[ ] Internet security – staying safe online<br>[ ] Buying a computer<br>[ ] Ten hints for silver surfers	http://downloads.bbc.co.uk/connect/volunteers_handbook.pdf<br>[ ] First click – beginners guide to the Internet<br>[ ] Give an hour and help someone take their first click online

Using the Internet for health care in Plymouth (PERQ2)

We want to hear from everybody, not just those who have used the Internet or are interested in using it.

My name is Professor Ray Jones and I am hoping that you will be able to help with this survey being carried out in Plymouth. The survey is about using the Internet for health care, but we want to hear from everybody regardless of whether or not you use it.

Completing the attached questionnaire should take about 15 minutes. Let me explain why we are doing this survey and why a response from your household is important.

Why are we doing this study?
A lot of people have found the Internet useful for their health but not everyone can access or wants to use it. Moreover, the NHS could improve services and save money (to be used in other parts of the NHS) by making better use of the Internet. Of course, not everyone wants to do things that way but it is important that everyone can have the opportunity. So our concern is about inequalities in being able to use the Internet for health.

We are concerned that not everyone has the opportunity to use the Internet for healthcare. At the moment we do not have a consistent way of assessing different people's opportunity and desire to use the Internet for healthcare. We need to make sure that any questionnaire 'works' for those who are not interested in the Internet as well as those who use it a lot. Therefore, to do that, we are developing and testing the questionnaire in this survey of Plymouth.

Who should complete this questionnaire?
We are hoping that one person in your household will complete and return the questionnaire in the reply paid envelope. As a way of making this a random choice, we would like the person with the next birthday to complete it, regardless of whether that person has any interest in health or the Internet. If that person needs help to complete it, we hope that someone in the household will help and tick the appropriate box on the first page.

Why should they complete it?
It will help us with our research and that research will eventually lead to a better understanding of inequalities in using the Internet for health. As an extra incentive:

- We will have a prize draw for each district in the study. The winner will receive an M&S voucher for £20. You have a good chance (about 1 in 60) of winning.
- You can receive a free printed copy of a guide to the Internet. If you are interested please tick the appropriate box on the last page of the questionnaire.

What will we do with your completed questionnaire?
We will be analysing the data to make sure that the questionnaire and survey methods 'work'. If you have any comments or suggestions about the survey please do let us know by writing in the space provided. The number on the questionnaire tells us which house it came from, to enable us to give the winner their M&S voucher. Otherwise the questionnaire is anonymous. We will keep the questionnaires in a locked cabinet until we have finished the survey. They will then be destroyed.

Thank you very much for your help. If you have any questions or comments before completing the questionnaire please contact me: Professor Ray Jones, Faculty of Health, Education and Society, Plymouth University, 3 Portland Villas, Plymouth. (ray.jones@plymouth.ac.uk)

Using the Internet for health care in Plymouth

We want to hear from <u>everybody</u>, not just those who have used the Internet or are interested in using it.

If this questionnaire is being completed by another member of the household on behalf of the adult with the next birthday, please ✓ tick this box [ ]

A. ABOUT YOU AND HEALTH INFORMATION AND SUPPORT

A1. Are you: Male [ ] Female [ ] A2. How old are you?.........................

A3. In the last three months have you ever (tick ✓all that apply):

Seen a doctor, nurse, or other health professional about your health [ ]
Asked a family member or friend something about your health [ ]
Phoned a helpline (e.g. NHS Direct, Samaritans, Diabetes UK) about your health [ ]
Read a book, or magazine to find something out about health [ ]
Used the Internet for something to do with your health [ ]
None of these [ ]

B. INTERNET USE FOR ANY PURPOSE

This section is about whether you have used the Internet, how often and where you use it.

B1. Have you <u>personally</u> used the Internet for any purpose in the last 3 months? Yes [ ] No [ ]
If yes, continue with **question B2 below.**
If no, go to **question C1 on the next page**

B2. Typically how often do you use the Internet (for any purpose)?

Many times a day [ ]
At least once a day [ ]
At least once a week [ ]
Less than once a week - every now and then [ ]

B3. What have you used the Internet for? Tick boxes in the first column for all the ways you have used the Internet for any reason, and tick boxes in the second column if you have used the Internet in that way for something related to your health.

	Have used the Internet for…..	
	Any purpose	For something health related
Browsing web pages (e.g. using Google)		
Email		
Internet telephony (eg Skype)		
Discussion forum		
Twitter		
Social network site (eg Facebook, Linked in)		
Watching videos		
Virtual World (e.g. Second Life)		

B4. Where and how have you accessed the Internet in the last 3 months? Tick all that apply.

Desktop / laptop computer at home [ ]
Desktop / laptop computer at work [ ]
Smart phone or mobile device such as iPAD [ ]
Desktop computer in a library or community centre [ ]
'Paid for' computer in an Internet café, shop, airport [ ]
Elsewhere [ ] (Where?...)

NOW PLEASE GO TO SECTION D

C. FOR PEOPLE WHO HAVE NOT USED THE INTERNET IN THE LAST THREE MONTHS
This section asks about whether you would like to use the Internet, if maybe you would like to use it for health related things, given help.
If you HAVE used the Internet in the last three months, go to section D.

C1. Have you ever used the Internet? (Tick one of the following)
I used to use it fairly frequently but not recently [ ]
I have only ever used it a few times and not recently [ ]
I have never used it [ ]

C2. Does your home have an Internet connected computer? Yes [ ] No [ ]

C3. Has anyone ever used the Internet for you, e.g. to find out something for you, or to buy something for you, or to contact someone on your behalf by email? Yes [ ] No [ ]

C4. If someone was able to help you, and it was easy, and it was cheap, would you make use of a home Internet connection? No [ ] Possibly [ ] Probably [ ] Yes [ ]

C5. In this table, tick the left hand column if you go there in a typical month, tick the middle column if you would use an Internet connected computer there (assuming that it was free, easy to use, and there was help to use it) for any purpose, and tick the right hand column if you would use it there to get health information.

	Tick if……		
Places you might go to in a typical month	You go there	You would use the Internet there if given the opportunity	You would use the Internet there for health information
A place of work			
A public library			
A community centre, or Age UK centre			
A place of worship			
Your doctor's practice			
Your local hospital			
The house of a family member			
A friend's house			
Other?.......			

If you have not used the Internet in the last three months, there is just one more page of questions that we would like you to complete. **Please go to section G on the last page.**

D. PROVISION OF INTERNET SERVICES
Answer this section if you have used the Internet for any purpose in the last three months.

D1. Does your home (tick ✓ one)……..
Have an Internet connection that is fast enough **<u>for you</u>** [ ]
Have an Internet connection that is slow for what **<u>you need</u>** [ ]
Have no Internet connection [ ]
Don’t know [ ]

D2. <u>Could</u> your home <u>currently</u> have a ‘fast enough (for you)’ Internet connection?
(Tick ✓ one only)
It already does [ ]
Probably, if I paid for it [ ]
I don’t think so (because of rurality, too much ‘traffic’, no provision etc) [ ]
Don’t know [ ]

D3. Does your General Practitioner (family doctor) have a website?
Yes I have looked at it [ ]
Yes I think so but I have not seen it [ ]
No [ ]
Don’t know [ ]

If your General Practitioner (GP) has a website:

D4. If you wanted, can you order a repeat prescription by email, or on your GP’s website? Yes [ ] No [ ] Don’t know [ ]

D5. If you wanted, can you see your own medical record online via your GP’s website? Yes [ ] No [ ] Don’t know [ ]

In the last three months, have you used the Internet trying to….

D6. Find <u>information</u> about health topics, services, treatments, advice etc.)?

Never tried [ ]
Tried, and found what I wanted most of the time [ ]
Tried, but not been able to find what I wanted [ ]
Can you say what topic?...

D7. <u>Contact</u> an organisation, or discussion forum, or other people, for some reasons connected with your health and been able to get what you wanted?

Never tried [ ]
Tried, and found what I wanted most of the time [ ]
Tried, but not been able to contact who I wanted [ ]
Can you say who/what organisation you were trying to contact?..........................

E. PERSONAL SKILLS AND CONFIDENCE IN USING THE INTERNET FOR HEALTH

This section is for people who have used the Internet in the last three months. It is about your own physical ability, knowledge, skills, interest, and emotional state in using the Internet.

E1. Do you have a permanent physical disability that makes using the Internet difficult?
No [ ]
Yes - makes use of the Internet very difficult [ ]
Yes - makes use of the Internet somewhat difficult [ ]
If Yes, please give details………………..

E2. Do you have, or have you had, a physical or mental condition that means makes using the Internet difficult just at the time when maybe it would be useful?
No [ ]
Yes – when I feel ill/bad, using the Internet is very difficult [ ]
Yes – when I feel ill/bad, using the Internet is somewhat difficult [ ]
If yes, please give details…………………….

E3. This question is a self-assessment of your **general** Internet skills, not necessarily concerned with health. In the following table, please read the 'task' and then tick one box to show if you think you could do that task.

I think I could…..	No	Probably	Yes
Book tickets for a film online and save a copy of the booking into a folder on your computer			
Search on Google with keyword 'Asthma', and open the first three sites found, to compare what they say			
Use a Google to find out what type of documents you need to apply for a new passport if yours was lost			
Compare the cost and convenience of a holiday using a 'package holiday' company to booking travel & accommodation separately			

E4. In general how confident are you in using the Internet **for health**, for example, knowing where and how to find helpful health resources, telling good quality from poor quality resources, or using the Internet to connect to a forum, or some other source of advice?

Circle a number between 1 (not at all confident) and 10 (totally confident).

Not confident………………………………………………………………..Totally confident									
1	2	3	4	5	6	7	8	9	10

F. SUPPORT FROM ANOTHER PERSON IN USING THE INTERNET FOR HEALTH

This third section is about whether you have, or could have if you wanted it, support from somebody in using the Internet for health.

F1. Has a doctor, nurse, or other health professional ever given **you** information (e.g. a web address) to help your use the Internet for your health? Yes [ ] No [ ]

F2. Near where you live or by phone or email, are people able to get **help using the Internet for health** from an organisation or society? (e.g. from the local library, Age UK, local authority, NHS, or University run service)

[ ] Don't know
[ ] Not that I am aware of
[ ] Yes, from …………………………………………………….

If **yes**, have **you** ever made use of such help? Yes [ ] No [ ]

F3. Have there ever been occasions when some help from somebody in using the Internet for health was or might have been useful? Yes [ ] No [ ]

If NO to F3, go to section G

F4. Do you have a family member or friend who could help you to use the Internet (for any purpose)?

No [ ]
Yes, there is someone I can ask quite easily [ ]
Yes, but they are not, or would not be very easy to ask [ ] Why is that?..............................

F5. **<u>If yes to question F4,</u>** would you feel OK about asking them to help you use the Internet for **<u>health</u>** purposes (to find information or to communicate with someone)?

Yes [ ]
No [ ] If no, why is that?..

F6. If you were to get support from family or friends in using the Internet for health would you have any concerns about disclosing information about your health?

[ ] No, not at all
[ ] Yes, depending on health issue. Please give details……………………………….
[ ] Definitely yes, please give details…………………………………………………….

F7. If you were to get support from an unknown person online or by phone in using the Internet for health and you were anonymous to them would you have any concerns about disclosing information about your health?

[ ] No, not really
[ ] Don't know
[ ] Yes, please give details………………………………………………………………….

F8. If you were to get support from family or friends in using the Internet for health would you have any concerns about being dependent on that person for help?

[ ] No, not really
[ ] Yes, please give details………………………………………………………………….

G. ECONOMIC CONSIDERATIONS IN USING THE INTERNET FOR HEALTH
This section asks about the cost to you of using the Internet for health

G1. How much do you think it costs **per month** to be connected to the Internet at home? (Please tick one box as your best guess even if you do not have the Internet).

£5	£10	£15	£20	£25	£30	£40	£50	£60	£70

G2. Please tick ✓ one box for each line to show whether you agree or disagree with each of the following statements, as they relate to you at the moment. They include statements about using the Internet but also about visiting your GP or hospital, for comparison.

For me………	Strongly agree	Agree	Disagree	Strongly disagree	Don't know
The monthly cost of home Internet is a major concern					
Mobile Internet access on smart phones and iPADS is expensive					
Getting to a public library to use the Internet does not cost much					
It costs me nothing, or very little, to get to see my GP					
It costs me nothing, or very little, to visit my nearest hospital					

H. What are your thoughts about the Internet and health?
This last section is an 'open section' for your comments. Do you have any thoughts about using the Internet for health? In particular, do you have any ideas on what could be done to help those who want access to the Internet for health, but do not have it either (a) because of lack of physical access, or (b) need to help or training, or (c) need for support from someone, or (d) because of the cost?

………………………………………………………………………………………………..

……………………………………………………………………………………………….

……………………………………………………………………………………………….

Thank you very much for your help in completing the questionnaire. If you send the questionnaire back in the prepaid envelope you will be entered into the prize draw for an Amazon voucher. The following six guides are all available on the Internet if you have access, but if you would like us to send you <u>one</u> free printed copy, please tick the appropriate box. Please tick one box only.

From Age UK	From the BBC
www.ageuk.org.uk/work-and-learning/technology-and-internet/<br>[ ] Making the most of the Internet<br>[ ] Internet security – staying safe online<br>[ ] Buying a computer<br>[ ] Ten hints for silver surfers	http://downloads.bbc.co.uk/connect/volunteers_handbook.pdf<br>[ ] First click – beginners guide to the Internet<br>[ ] Give an hour and help someone take their first click online

Using the Internet for health care in the PL postcode area (PERQ3)

We want to hear from all adults, not just those who have used the Internet or are interested in using it.

My name is Professor Ray Jones and I am hoping that you will be able to help with this survey being carried out in the PL postcode area. The survey is about using the Internet for health care, but we want to hear from all adults regardless of whether or not you use it. We particularly want to hear from you if you have never used the Internet and do not know much about it, as you often get 'missed out'.

Completing the attached questionnaire should take about 15 minutes. This letter explains why we are doing this survey and why a response from your household is important.

Why are we doing this study?
We know many people find useful health information on the Internet, but some people can't access it. Not everyone wants to use it, but it is important that everyone can if they want to. This could also help the NHS to save money which can then be used to fund other services. We want to do a survey to find out whether people can use the Internet for health care if they want to, and whether anything is hindering them. To do this we need to test a questionnaire and see if it works for everyone, whether or not they are interested in the Internet.

Who should complete this questionnaire?
We are hoping that one adult (aged 16+) in your household will complete and return the questionnaire in the reply paid envelope. As a way of making this a random choice, we would like the adult with the next birthday to complete it, regardless of whether that person has any interest in health or the Internet.

Why should they complete it?
It will help us with our research and that research will eventually lead to a better understanding of inequalities in using the Internet for health. As an extra incentive:

- We will have a prize draw for each district in the study. The winner will receive an M&S voucher for £20. You have a good chance (about 1 in 60) of winning.
- You can receive a free printed copy of a guide to the Internet. If you are interested please tick the appropriate box on the last page of the questionnaire.

What will we do with your completed questionnaire?
We will be analysing the data to make sure that the questionnaire and survey methods 'work'. If you have any comments or suggestions about the survey please do let us know by writing in the space provided. The number on the questionnaire tells us which house it came from, to enable us to give the winner their M&S voucher. Otherwise the questionnaire is anonymous. For that reason, once you have posted the questionnaire we would not be able to withdraw your data from the survey. We will keep the questionnaires in a locked cabinet until we have finished the survey. They will then be destroyed.

Thank you very much for your help. If you have any questions or comments before completing the questionnaire please contact me: Professor Ray Jones, Faculty of Health, Education and Society, Plymouth University, 3 Portland Villas, Plymouth. (ray.jones@plymouth.ac.uk)

Using the Internet for health care in the PL postcode area

We want to hear from <u>everybody</u>, not just those who have used the Internet or are interested in using it.

If this questionnaire is being completed by another member of the household on behalf of the adult with the next birthday, please tick this box [ ]

A. ABOUT YOU AND HEALTH INFORMATION AND SUPPORT

A1. Are you: Male [ ] Female [ ] A2. How old are you?........................

A3. In the last three months have you (tick ✓all that apply):

Seen a doctor, nurse, or other health professional about your health [ ]
Asked a family member or friend something about your health [ ]
Phoned a helpline (e.g. NHS Direct, Samaritans, Diabetes UK) about your health [ ]
Read a book, or magazine to find something out about your health [ ]
Used the Internet for something to do with your health [ ]
None of the above [ ]

B. INTERNET USE FOR ANY PURPOSE

This section is about whether you have used the Internet, how often and where you use it.

B1. Have you <u>personally</u> used the Internet for any purpose in the last 3 months?

Yes [ ] No [ ] If yes, continue with **question B2 below.**
If no, go to **question C1 on the next page**

B2. Typically how often do you use the Internet (for any purpose)?

Many times a day [ ]
At least once a day [ ]
At least once a week [ ]
Less than once a week - every now and then [ ]

B3. What have you used the Internet for? Tick boxes in the first column for all the ways you have used the Internet for any purpose, and tick boxes in the second column for all the ways you have used the Internet for something related to your health.

	Have used the Internet for…..	
	Any purpose	For something health related
To find information (e.g. using Google)		
Email		
Internet telephony (eg Skype)		
Discussion forum		
Twitter		
Social network site (eg Facebook, Linked in)		
Watching videos (e.g. YouTube)		
Virtual World (e.g. Second Life)		

B4. Where and how have you accessed the Internet in the last 3 months? Tick all that apply.

Desktop / laptop computer at home [ ]
Desktop / laptop computer at work [ ]
Smart phone or mobile device (e.g. iPhone, iPAD) [ ]
Desktop computer in a library or community centre [ ]
'Paid for' computer in an Internet café, shop, airport [ ]
Elsewhere [ ] (Where?...)

NOW PLEASE GO TO SECTION D

C. FOR PEOPLE WHO HAVE NOT USED THE INTERNET IN THE LAST THREE MONTHS
This section asks about if you have EVER used it, whether you would like to use the Internet, if maybe you would like to use it for health related things, given help.
If you HAVE used the Internet in the last three months, go to section D.

C1. Have you EVER used the Internet (for any purpose)? (Tick one of the following)
I used to use it fairly often but not recently [ ]
I have only ever used it a few times and not recently [ ]
I have never used it [ ]

C2. Does your home have an Internet connected computer? Yes [ ] No [ ]

C3. Has anyone ever used the Internet for you (e.g. to find out something for you, or to buy something for you, or to contact someone on your behalf by email)? Yes [ ] No [ ]

C4. If someone was able to help you, and it was easy, and it was cheap, would you use a home Internet connection? No [ ] Possibly [ ] Probably [ ] Yes [ ]

C5. In this table,
i. tick boxes in the left hand column for all the places you go to in a typical month

ii. tick boxes in the middle column for all the places you would use an Internet connected computer (assuming that it was there, free, easy to use, and there was help to use it) for any purpose

iii. tick boxes in the right hand column for all the places you would be prepared to use an Internet connected computer to get health information.

	Tick if……		
Places you might go to in a typical month	i. You go there	ii. You would use the Internet there if given the opportunity	iii. You would use the Internet there for health information
A place of work			
A public library			
A community centre, or Age UK centre			
A place of worship			
Your doctor's practice			
Your local hospital			
The house of a family member			
A friend's house			
Other?.......			

If you have not used the Internet in the last three months, there is just one more page of questions that we would like you to complete.

Please go to sections G and H on the last page.

D. ACCESS TO INTERNET SERVICES
Answer this section if you have used the Internet **for any purpose** in the last three months.

D1. Does your home (tick ✓ one)……..
- Have an Internet connection that is fast enough **for what you need** [ ]
- Have an Internet connection that is slow for what **you need** [ ]
- Have no Internet connection [ ]
- Don't know [ ]

D2. If you do not have an Internet connection, or one that is good enough, why is that?
- I would need to pay more [ ]
- I live in a rural area and there is no good connection to my home [ ]
- My local server is congested and unreliable [ ]
- My Internet provider does not offer a faster connection [ ]
- Don't know [ ]

D3. Does your General Practitioner (family doctor) have a website (e.g. that you might find by Google)?
- Yes I have looked at it [ ]
- Yes I think so but I have not seen it [ ]
- No [ ]
- Don't know [ ]

If your General Practitioner (GP) has a website:

D4. If you wanted, can you order a repeat prescription by email, or on your GP's website?
Yes [ ]   No [ ]   Don't know [ ]

D5. If you wanted, can you see your own medical record online via your GP's website?
Yes [ ]   No [ ]   Don't know [ ]

D6 In the last three months, have you used the Internet trying to find information about health topics, services, treatments, advice etc.)?

- Never tried [ ]
- Tried, and found what I wanted most of the time [ ]
- Tried, but not been able to find what I wanted [ ]
  - Can you say what topic?...

D7. In the last three months, have you used the Internet trying to contact an organisation, or discussion forum, or other people, for some reasons connected with your health and been able to get what you wanted?

- Never tried [ ]
- Tried, and found what I wanted most of the time [ ]
- Tried, but not been able to contact who I wanted [ ]
  - Can you say who/what organisation you were trying to contact?..........................

E. PERSONAL SKILLS AND CONFIDENCE IN USING THE INTERNET FOR HEALTH

This section is for people who have used the Internet in the last three months. It is about your own physical ability, knowledge, skills, and interest in using the Internet.

E1. Do you have a long term disability that makes using the Internet difficult?

No [ ]
Yes - makes use of the Internet very difficult [ ]
Yes - makes use of the Internet somewhat difficult [ ]
If Yes, please give details………………..

E2. This question is a self-assessment of your **general** Internet skills, not necessarily concerned with health. In the following table, please read the 'task' and then tick one box to show if you think you could do that task.

I think I could…..	No	Maybe	Yes
Book tickets for a film online and save a copy of the booking into a folder on your computer			
Search on Google with keyword 'Asthma', and open the first three sites found, to compare what they say			
Use Google to find out what type of documents you need to apply for a new passport if yours was lost			
Compare the cost and convenience of a holiday using a 'package holiday' company to booking travel & accommodation separately			

E3. In general how confident are you in using the Internet **for health**, for example, knowing where and how to find helpful health resources, telling good quality from poor quality resources, or using the Internet to join a forum, or some other source of advice?

Circle a number between 1 (not at all confident) and 10 (totally confident).

Not confident………………………………………………………………..Totally confident									
1	2	3	4	5	6	7	8	9	10

F. SUPPORT FROM ANOTHER PERSON IN USING THE INTERNET FOR HEALTH
This third section is about whether you have, or could have if you wanted it, support from somebody in using the Internet for health.

F1. Has a doctor, nurse, or other health professional ever given you information (e.g. a web address) to help you use the Internet for your health? Yes [ ] No [ ]

F2. If you wanted help using the internet, could you find it near where you live, or by phone or email?
(e.g. from the local library, Age UK, local authority, NHS, or University run service)
[ ] Don't know
[ ] Not that I am aware of
[ ] Yes, from ……………………………………………………………

If yes, have you ever made use of such help? Yes [ ] No [ ]

F3. Have there ever been times when help from somebody in using the Internet for health was or might have been useful? Yes [ ] No [ ]

If NO to F3, go to section G

F4. Do you have a family member or friend who could help you to use the Internet (for any purpose)?
No [ ]
Yes, there is someone I can ask quite easily [ ]
Yes, but they are not, or would not be very easy to ask [ ] Why is that?..............................

F5. **If yes to question F4,** would you feel OK about asking them to help you use the Internet for **health** purposes (to find information or to communicate with someone)?
Yes [ ]
No [ ] If no, why is that?..

F6. If you were to get support from an unknown person online or by phone in using the Internet for health, and you were anonymous to them, would you have any concerns about disclosing information about your health?
[ ] No, not really
[ ] Don't know
[ ] Yes, please give details………………………………………………………………….

G. ECONOMIC CONSIDERATIONS IN USING THE INTERNET FOR HEALTH

This last section asks about the cost to you of using the Internet for health. Please answer even if you have never used the Internet.

G1. Please read each of the statements and tick one box for each to show whether you agree or disagree, as they relate to you at the moment.

For me………	Strongly agree	Agree	Disagree	Strongly disagree	Don't know
The monthly cost of home Internet is a major concern					
Mobile Internet access on smart phones and iPADS is expensive					
Getting to a public library to use the Internet does not cost much					
It costs me nothing, or very little, to get to see my GP					
It costs me nothing, or very little, to visit my nearest hospital					

H. OVERALL VIEWS ABOUT USING THE INTERNET FOR HEALTH

This last section asks about the factors most likely to reduce your use of the Internet for health, and for your views. Please answer even if you have never used the Internet.

H1. Which **one** thing is most important in stopping you using the Internet, or using it more, for your health?
[ ] NEED: I have no need for health information
[ ] PHYSICAL ACCESS: I have problems getting access to a good Internet connection
[ ] PERSONAL REASONS: I have no interest in the Internet or I don't understand the Internet that much
[ ] SUPPORT: I do not have anyone to help me use the Internet
[ ] ECONOMIC: I think that using the Internet is too expensive for me

H2. Lastly an open question. Do you have any thoughts about using the Internet for health? What could be done to help those who want access to the Internet for health?
…………………………………………………………………………………………………….

…………………………………………………………………………………………………….

…………………………………………………………………………………………………….

Thank you very much for your help in completing the questionnaire. The following six guides are all available on the Internet if you have access, but if you would like us to send you one free printed copy, please tick the appropriate box. Please tick one box only.

From Age UK	From the BBC
www.ageuk.org.uk/work-and-learning/technology-and-internet/<br>[ ] Making the most of the Internet<br>[ ] Internet security – staying safe online<br>[ ] Buying a computer<br>[ ] Ten hints for silver surfers	http://downloads.bbc.co.uk/connect/volunteers_handbook.pdf<br>[ ] First click – beginners guide to the Internet<br>[ ] Give an hour and help someone take their first click online

Using the Internet for health care in the PL postcode area (PERQ4)

We want to hear from all adults, not just those who have used the Internet or are interested in using it.

My name is Professor Ray Jones and I am hoping that you will be able to help with this survey being carried out in the PL postcode area. The survey is about using the Internet for health care, but we want to hear from all adults regardless of whether or not you use it. We particularly want to hear from you if you have never used the Internet and do not know much about it, as you often get 'missed out'.

Completing the attached questionnaire should take about 15 minutes. This letter explains why we are doing this survey and why a response from your household is important.

Why are we doing this study?
We know many people find useful health information on the Internet, but some people can't access it. Not everyone wants to use it, but it is important that everyone can if they want to. This could also help the NHS to save money which can then be used to fund other services. We want to do a survey to find out whether people can use the Internet for health care if they want to, and whether anything is hindering them. To do this we need to test a questionnaire and see if it works for everyone, whether or not they are interested in the Internet.

Who should complete this questionnaire?
We are hoping that one adult (aged 16+) in your household will complete and return the questionnaire in the reply paid envelope. As a way of making this a random choice, we would like the adult with the next birthday to complete it, regardless of whether that person has any interest in health or the Internet.

Why should they complete it?
It will help us with our research and that research will eventually lead to a better understanding of inequalities in using the Internet for health. As an extra incentive:

- We will have a prize draw for each district in the study. The winner will receive an M&S voucher for £20. You have a good chance (about 1 in 60) of winning.
- You can receive a free printed copy of a guide to the Internet. If you are interested please tick the appropriate box on the last page of the questionnaire.

What will we do with your completed questionnaire?
We will be analysing the data to make sure that the questionnaire and survey methods 'work'. If you have any comments or suggestions about the survey please do let us know by writing in the space provided. The number on the questionnaire tells us which house it came from, to enable us to give the winner their M&S voucher. Otherwise the questionnaire is anonymous. For that reason, once you have posted the questionnaire we would not be able to withdraw your data from the survey. We will keep the questionnaires in a locked cabinet until we have finished the survey. They will then be destroyed.

Thank you very much for your help. If you have any questions or comments before completing the questionnaire please contact me: Professor Ray Jones, Faculty of Health, Education and Society, Plymouth University, 3 Portland Villas, Plymouth. (ray.jones@plymouth.ac.uk)

Using the Internet for health care in the PL postcode area

We want to hear from <u>everybody</u>, not just those who have used the Internet or are interested in using it.

If this questionnaire is being completed by another member of the household on behalf of the adult with the next birthday, please tick this box [ ]

A. ABOUT YOU AND HEALTH INFORMATION AND SUPPORT

A1. Are you: Male [ ] Female [ ] A2. How old are you?........................

A3. In the last three months have you (tick ✓all that apply):

Seen a doctor, nurse, or other health professional about your health [ ]
Asked a family member or friend something about your health [ ]
Phoned a helpline (e.g. NHS Direct, Samaritans, Diabetes UK) about your health [ ]
Read a book, or magazine to find something out about your health [ ]
Used the Internet for something to do with your health [ ]
None of the above [ ]

B. INTERNET USE FOR ANY PURPOSE

This section is about whether you have used the Internet, how often and where you use it.

B1. Have you <u>personally</u> used the Internet for any purpose in the last 3 months?

Yes [ ] No [ ] If yes, continue with **question B2 below.**
If no, go to **question C1 on the next page**

B2. Typically how often do you use the Internet (for any purpose)?

Many times a day [ ]
At least once a day [ ]
At least once a week [ ]
Less than once a week - every now and then [ ]

B3. What have you used the Internet for? Tick boxes in the first column for all the ways you have used the Internet for any purpose, and tick boxes in the second column for all the ways you have used the Internet for something related to your health.

	Have used the Internet for…..	
	Any purpose	For something health related
To find information (e.g. using Google)		
Email		
Internet telephony (eg Skype)		
Discussion forum		
Twitter		
Social network site (eg Facebook, Linked in)		
Watching videos (e.g. YouTube)		
Virtual World (e.g. Second Life)		

B4. Where and how have you accessed the Internet in the last 3 months? Tick all that apply.

Desktop / laptop computer at home [ ]
Desktop / laptop computer at work [ ]
Smart phone or mobile device (e.g. iPhone, iPAD) [ ]
Desktop computer in a library or community centre [ ]
'Paid for' computer in an Internet café, shop, airport [ ]
Elsewhere [ ] (Where?...)

NOW PLEASE GO TO SECTION D

C. FOR PEOPLE WHO HAVE NOT USED THE INTERNET IN THE LAST THREE MONTHS
This section asks about if you have EVER used it, whether you would like to use the Internet, if maybe you would like to use it for health related things, given help.
If you HAVE used the Internet in the last three months, go to section D.

C1. Have you EVER used the Internet (for any purpose)? (Tick one of the following)
I used to use it fairly often but not recently [ ]
I have only ever used it a few times and not recently [ ]
I have never used it [ ]

C2. Do you have a long term disability that would make using a computer difficult?
No [ ]
Yes – it would make using a computer very difficult [ ]
Yes – it would make using a computer somewhat difficult [ ]
If Yes, please give details……………….

C3. Does your home have an Internet connected computer? Yes [ ] No [ ]

C4. As far as you know do any of your neighbours have Internet access? Yes [ ] No [ ] Don't know [ ]

C5. Has anyone ever used the Internet for you (e.g. to find out something for you, or to buy something for you, or to contact someone on your behalf by email)? Yes [ ] No [ ]

C6. If someone was able to help you, would you 'have a go' at using the Internet?
No, it's really not for me [ ] Possibly [ ] Probably [ ] Yes [ ]

C7. If you would 'have a go' using the Internet, do you have someone (e.g. family, friend, neighbour) who could help you?
No [ ]
Yes, there is someone I can ask easily [ ]
Yes, but they are not , or would not be very easy to ask [ ] Why is that?.....................................

C8. If someone was able to help you, and it was easy, and it was cheap, would you use a home Internet connection? No [ ] Possibly [ ] Probably [ ] Yes [ ]

C9. If there were Internet connected computers available at some place (such as those listed below) that you go to, and they were free to use, easy to use, and there was help there to use them for any purpose, would you consider using them? No [ ] Possibly [ ] Probably [ ] Yes [ ]

C10. If you answered possibly, probably, or yes, to question C9, in this list tick those places where you might be prepared to use the Internet for health. If none of these please tick last row.

A place of work	
A public library	
A community centre, or Age UK centre	
A place of worship	
Your doctor's practice	
Your local hospital	
The house of a family member	
A friend's house	
Other? (say where).........	
**NONE of these**	

If you have not used the Internet in the last three months,
Please go to sections G and H on the last page.

D. ACCESS TO INTERNET SERVICES
Only answer this section if you have used the Internet **for any purpose** in the last three months. If you have **not** used the Internet turn over to the last page section G.

D1. Does your home (tick ✓ one)……..
Have an Internet connection that is fast enough **for what you need** [ ] (Go to D3).
Have an Internet connection that is slow for what **you need** [ ]
Have no Internet connection [ ]
Don't know [ ]

D2. If you do not have an Internet connection, or slow for what you need, why is that? (tick ✓ one)
I would need to pay more [ ]
I live in a rural area and there is no good connection to my home [ ]
My local server is congested and unreliable [ ]
My Internet provider does not offer a faster connection [ ]
Don't know [ ]

D3. Does your General Practitioner (family doctor) have a website (e.g. that you might find by Google)?
Yes I have looked at it [ ]
Yes I think so but I have not seen it [ ]) (Go to D6)
No [ ])
Don't know [ ])

If your General Practitioner (GP) has a website AND you have looked at it:
D4. If you wanted, can you order a repeat prescription by email, or on your GP's website?
Yes [ ] No [ ] Don't know [ ]

D5. If you wanted, can you see your own medical record online via your GP's website?
Yes [ ] No [ ] Don't know [ ]

D6 In the last three months, have you used the Internet trying to find information about health topics, services, treatments, advice etc.)?

Never tried [ ]
Tried, and found what I wanted most of the time [ ]
Tried, but not been able to find what I wanted [ ]
Can you say what topic?...

D7. In the last three months, have you used the Internet trying to contact an organisation online, or discussion forum, or other people, for some reasons connected with your health and been able to get what you wanted?

Never tried [ ]
Tried, and found what I wanted most of the time [ ]
Tried, but not been able to contact who I wanted [ ]
Can you say who/what organisation you were trying to contact?..........................

E. PERSONAL SKILLS AND CONFIDENCE IN USING THE INTERNET FOR HEALTH

This section is about whether you have or could have if you wanted it support from somebody in using the Internet for health. Only answer this section if you have used the Internet **for any purpose** in the last three months. If you have **not** used the Internet turn over to the last page section G.

E1. Do you have a long term disability that makes using the Internet difficult?

No [ ]
Yes - makes use of the Internet very difficult [ ]
Yes - makes use of the Internet somewhat difficult [ ]
If Yes, please give details……………….

E2. This question is a self-assessment of your **general** Internet skills, not necessarily concerned with health. In the following table, please read the 'task' and then tick one box to show if you think you could do that task.

I think I could…..	No	Maybe	Yes
Book tickets for a film online and save a copy of the booking into a folder on your computer			
Search on Google with keyword 'Asthma', and open the first three sites found, to compare what they say			
Use Google to find out what type of documents you need to apply for a new passport if yours was lost			
Compare the cost and convenience of a holiday using a 'package holiday' company to booking travel & accommodation separately			

E3. In general how confident are you in using the Internet **for health**, for example, knowing where and how to find helpful health resources, telling good quality from poor quality resources, or using the Internet to join a forum, or some other source of advice?

Circle a number between 1 (not at all confident) and 10 (totally confident).

Not confident………………………………………………………………..Totally confident									
1	2	3	4	5	6	7	8	9	10

F. SUPPORT FROM ANOTHER PERSON IN USING THE INTERNET FOR HEALTH
Only answer this section if you have used the Internet **for any purpose** in the last three months.
If you have **not** used the Internet turn over to the last page section G.
This section is about whether you have, or could have if you wanted it, support from somebody in using the Internet for health.

F1. Has a doctor, nurse, or other health professional ever given you information (e.g. a web address) to help you use the Internet for your health? Yes [ ] No [ ]

F2. If you, or someone in your household, wanted help using the Internet, could you find it near where you live, or by phone or email? (e.g. from local library, Age UK, local authority, NHS, or University).
[ ] Don't know
[ ] Not that I am aware of
[ ] Yes, from ………………………………………………………

If yes, have you ever made use of such help? Yes [ ] No [ ]

F3. Have there ever been times when help from somebody in using the Internet for health was or might have been useful for you? Yes [ ] No [ ]

If NO to F3, go to section G

F4. Do you have a family member or friend who could help you to use the Internet (for any purpose)?
No [ ]
Yes, there is someone I can ask quite easily [ ]
Yes, but they are not, or would not be very easy to ask [ ] Why is that?..............................

F5. **If yes to question F4,** would you feel OK about asking them to help you use the Internet for **health** purposes (to find information or to communicate with someone)?
Yes [ ]
No [ ] If no, why is that?...

F6. If you were to get support from an unknown person online or by phone in using the Internet for health, and you were anonymous to them, would you have any concerns about disclosing information about your health?
[ ] No, not really
[ ] Don't know
[ ] Yes, please give details…………………………………………………………………

G. ECONOMIC CONSIDERATIONS IN USING THE INTERNET FOR HEALTH

This last section asks about the cost to you of using the Internet for health. Please answer even if you have never used the Internet.

G1. Please read each of the statements and tick one box for each to show whether you agree or disagree, as they relate to you at the moment.

For me………	Strongly agree	Agree	Disagree	Strongly disagree	Don't know
The monthly cost of home Internet is a major concern					
Mobile Internet access on smart phones and iPADS is expensive					
Getting to a public library to use the Internet does not cost much					
It costs me nothing, or very little, to get to see my GP					
It costs me nothing, or very little, to visit my nearest hospital					

H. OVERALL VIEWS ABOUT USING THE INTERNET FOR HEALTH

This last section asks about the factors most likely to reduce your use of the Internet for health, and for your views. Please answer even if you have never used the Internet.

H1. Which **one** statement best sums up how you feel about using the Internet for health? None of them may be exactly right, but try to choose one and then you can qualify your answer in the space in H2.
[ ] I have no need for health information
[ ] I have no interest in using the Internet
[ ] I would use the Internet more for health if I could get a good Internet connection
[ ] I don't understand the Internet that much
[ ] I would use the Internet more for health if I could get someone to help me
[ ] I would use the Internet more for health if money were no object
[ ] I have or would use the Internet for health and have no real barriers to that use

H2. Lastly an open question. Do you have any thoughts about using the Internet for health? What could be done to help those who want access to the Internet for health? (Also use this space if you want to qualify your answer to H1).
…………………………………………………………………………………………………….

…………………………………………………………………………………………………….

…………………………………………………………………………………………………….

Thank you very much for your help in completing the questionnaire. The following six guides are all available on the Internet if you have access, but if you would like us to send you one free printed copy, please tick the appropriate box. Please tick one box only.

From Age UK	From the BBC
www.ageuk.org.uk/work-and-learning/technology-and-internet/<br>[ ] Making the most of the Internet<br>[ ] Internet security – staying safe online<br>[ ] Buying a computer<br>[ ] Ten hints for silver surfers	http://downloads.bbc.co.uk/connect/volunteers_handbook.pdf<br>[ ] First click – beginners guide to the Internet<br>[ ] Give an hour and help someone take their first click online

4. VARIABLES FROM THE QUESTIONNAIRE AND THEIR USES FOR SUB-SCALES

Variable	Scores in, or reports as	Crosschecks with	Face validity, demographics, and other notes.
**A. About you and health information and support**			
A1			Demographics
A2			Demographics
A3	Need, Neednonweb	B1	Modifies provision
**B. Internet use for any purpose**			
B1		A3, B2, B4, C	Face Validity and section choice
B2	Frequency	B1	
B3	Range, rangehealth	D7	
B4	Provisioninternet, ubiquity	B1, D1,	
**C. For people who have not used the Internet in the last three months**			
C1	Lapsed	B1	Face validity check
C2	Disability	B1	
C3	Provision	B1	
C4	Provision	B1	
C5	Support		
C6	Personal		
C7	Support		
C8	Personal		
C9	Personal		
C10	Places		Explains C9
**D. Access to Internet Services**			
D1	Provision, Economic	B4, D2	
D2	Provision, Economic	D1	
D3	Provision		
D4	Provision		
D5	Provision		
D6	Provision		
D7	Provision	B3	
**E. Personal skills and confidence in using the Internet for health**			
E1	Disability		Skills used to modify personal. Personal then used to create modified Support score.
E2	Skills, Personal		
E3	Personal		
**F. Support from another person in using the Internet for health**			
F1	Support	UsedInternet	
F2	Support		
F3	Support		
F4	Support		
F5	Support		

F6	Support		
**G. Economic considerations in using the Internet for health**			
G1	Economic		
**H. Overall views about using the Internet for health**			
H1	Readiness		Check against sub-scales
H2	Comments		
**Final Box**			
INFO	Info needs		

5. SPSS syntax

```
comment data is entered using variable names as on the questionnaire A1, A2, A3, B1, B2 etc.
comment where respondents choose one answer, values are 1,2,3,4 etc.
comment where a question can have multiple answers (eg B3) then each (sub) variable has value 0 or 1.
comment this syntax file produces five variables each with scores 0-9 (0= worst , 9=best).
comment       NEED: motivation/need for health information.
comment       PROVISION: physical access to the Internet.
comment This has 2 components (provisioninternet 4 points) and provisionhlth GP provision (3.5 points), condition specific information and support (1.5 point).
comment Provisionhlth is scored out of 3.5 if no need/motivation for health information).
comment       PERSONAL: capability.
comment       SUPPORT: inter-personal support.
comment       ECONOMIC.
comment It also produces ‘compacted’ versions of provision, personal, economic, support scores on a scale of 0-4 (perq/2 and truncated).
comment.
compute disability=0.
compute economic=0.
compute provisioninternet=0.
compute homeormobileaccess=0.
comment CONSISTENCY CHECKS SETS QUERY INDICATORS******************************************.
comment       CONSISTENCY: If A3e=1 b1=2, ie if used Internet in last 3 months for health but ticked no to use it personally in last 3 months on B2, set QUERY =1.
comment CONSISTENCY the above is possible if they have got somebody else to use the Internet for them.
if a3e=1 and b1=2 query1=1.
if (missing(d1) or d1=3) and (b4a=1 and b4c=1) query10=1.
comment the above is if someone seems to be using mobile access for home Internet.
comment CONSISTENCY if someone has no Internet connection in section D but looks as if they do in section B.
if d1=3 and b4a=1 query4=1.
comment if answered that using Internet but then not entered how often query=2.
if b1=1 and missing (b2) query2=1.
comment if say not used Internet but still completed section B set query =7. Need to be checked to see how to handle data.
comment if section C completed, just 'blank' the rest of section B.
if b1=2 and b2>0 query7=1.
if b1=2 and (b4a>0 or b4b>0 or b4c>0 or b4d>0 or b4e>0 or b4f>0) query7=1.
comment if section C completed but question B1 not answered or B1=1 query =8.
if (missing(b1) or b1=1) and c1>0 query8=1.
comment CONSISTENCY if home access fine (d1) they should not have answered d2.
if d1=1 and d2>0 query5=1.
comment CONSISTENCY if tried to contact someone should have shown up on using email, discussion forum, etc in section B.
```

```
if (d7>1 and b3b2=0 and b3d2=0 and b3e2=0) query6=1.
if missing(f1) and missing (f2) and missing (f3) and usedinternet=1 query11=1.
comment TIDY UP DATA ****************************************************************************************.
comment , zeros sections D, E and F for non Internet users.
if b1=2 d1=0.
if b1=2 d2=0.
if b1=2 d3=0.
if b1=2 d4=0.
if b1=2 d5=0.
if b1=2 d6=0.
if b1=2 d7=0.
if b1=2 e1=0.
if b1=2 e2a=0.
if b1=2 e2b=0.
if b1=2 e2c=0.
if b1=2 e2d=0.
if b1=2 e3=0.
if b1=2 f1=0.
if b1=2 f2=0.
if b1=2 f3=0.
if b1=2 f4=0.
if b1=2 f5=0.
if b1=2 f6=0.
if f3=2 f4=0.
if f3=2 f5=0.
if f3=2 f6.0.
comment ****************************************************************************************.
comment NEED (score=10) depending on recent use of health professionals and/or seeking for health information.
comment neednonweb is same but omitting A3e (use of Internet).
comment.
compute need=0.
if a3a=1 need=need+2.
if a3b=1 need=need+2.
if a3c=1 need=need+2.
if a3d=1 need=need+2.
if a3e=1 need=need+2.
comment if ticked "none of the above" then zero A3 regardless of whether ticked items above.
If a3f=1 need=0.
if need=0 and missing (a3f) need=99.
compute neednonweb=need.
if a3e=1 neednonweb=need-2.
comment need is missing if nothing ticked in asnwer to question A3.
comment ****************************************************************************************.
comment B INTERNET USE FOR ANY PURPOSE.
```

```
comment.
comment section B summary variables.
compute usedinternet=99.
comment 99=missing value.
if b1=1 usedinternet=1.
if b1=2 usedinternet=0.
compute frequency=usedinternet*(5-b2).
compute rangeuseany=0.
compute rangeusehlth=0.
if b3a1=1 rangeuseany=rangeuseany+1.
if b3b1=1 rangeuseany=rangeuseany+1.
if b3c1=1 rangeuseany=rangeuseany+1.
if b3d1=1 rangeuseany=rangeuseany+1.
if b3e1=1 rangeuseany=rangeuseany+1.
if b3f1=1 rangeuseany=rangeuseany+1.
if b3g1=1 rangeuseany=rangeuseany+1.
if b3h1=1 rangeuseany=rangeuseany+1.
compute rangeuseany4=rnd(rangeuseany/2).
compute internethealthcomm=0.
compute usedinternethealth=0.
if b3a2=1 rangeusehlth=rangeusehlth+1.
if b3a2=1 usedinternethealth=1.
if b3b2=1 rangeusehlth=rangeusehlth+1.
if b3b2=1 internethealthcomm=1.
if b3c2=1 rangeusehlth=rangeusehlth+1.
if b3c2=1 internethealthcomm=1.
if b3d2=1 rangeusehlth=rangeusehlth+1.
if b3d2=1 internethealthcomm=1.
if b3e2=1 rangeusehlth=rangeusehlth+1.
if b3e2=1 internethealthcomm=1.
if b3f2=1 rangeusehlth=rangeusehlth+1.
if b3f2=1 internethealthcomm=1.
if b3g2=1 rangeusehlth=rangeusehlth+1.
if b3g2=1 usedinternethealth=1.
comment videos not added to internethealthcomm.
if b3h2=1 rangeusehlth=rangeusehlth+1.
if b3h2=1 internethealthcomm=1.
if internethealthcomm=1 usedinternethealth=1.
compute rangeusehlth4=rnd(rangeusehlth/2).
comment CONSISTENCY if b4a=1 (home access) d1 should =1 or 2, if not QUERY=3.
comment CONSISTENCY if d1=3 possible that lost internet connection, if d1=4 could be that does not
understand question of 'speed' - but worth checking questionnaires.
if b4a=1 and (missing (d1) or d1>2) query3=1.
compute ubiquity=0.
comment a (work), c (mobile) add 8, b (work) add 4, d,e,f (other) add 1 each.
```

```
comment ubiquity ranges from 1-23 with 0 as missing value (no place entered).
comment Anyone with score 8 or more has either home or mobile access.
if b4f=1 ubiquity=ubiquity+1.
if b4e=1 ubiquity=ubiquity+1.
if b4d=1 ubiquity=ubiquity+1.
if b4b=1 ubiquity=ubiquity+4.
if b4a=1 ubiquity=ubiquity+8.
if b4c=1 ubiquity=ubiquity+8.
if (Ubiquity>0) and (ubiquity<5) ubiquity5=1.
if (ubiquity>4) and (ubiquity<10) ubiquity5=2.
if (ubiquity>10) and (ubiquity<16) ubiquity5=3.
if (ubiquity>15) and (ubiquity<20) ubiquity5=4.
if (ubiquity>19) ubiquity5=5.
comment this (ubiquity) gives more weight to home and smart phone/tablet compared to work or
someone else's PC.
comment PROVISION ***********************************.
comment should be answering about provision in section D.
comment ******************************************************************************************************.
comment SECTION C non Internet Users******************************************************************************.
comment check to see if section C completed by Internet user, if so set query=9..
if (c1>0 OR C2>0 OR C3>0 or c4>0) and b1=1 query9=1.
comment.
comment disability=0, if C2=2 disability=disability+2, if c2=3 disability=disability+1.
if c2=2 disability=disability+2.
if c2=3 disability=disability+1.
comment PROVISION If the house has Internet (C3=1) provision =1.5.
comment if house has no internet (C3=2) and neighbours have Internet (C4=1) provision=1 (as it seems
as if the house could have Internet if the respondent wanted it).
if c3=1 provisioninternet=1.5.
if c3=1 homeormobileaccess=1.
if (c3=2 and c4=1) provisioninternet=1.
comment SUPPORT if C5=1 +2, if C7=2 +2, if C7=3 +1.
comment SUPPORT has maximum of 4 for non internet users (on the assumption that if their support
was effective they would be using the Internet!).
compute support=0.
if c5=1 support=support+2.
if c7=2 support=support+2.
if c7=3 support=support+1.
comment PERSONAL= (C6-1) + (C8-1) + (C9-1) /3.
comment each question is an indication of how much they would be prepared to have a go at the
Internet and is scored up to three, so the maximum score is 9/3=3. .
comment c10 provides indication of suitable places if not the home, but not used to add to score.
compute personal= ((c6-1)+(c8-1)+(c9-1))/3.
comment *******************************************************************************************************.
comment SECTION D PROVISION.
```

```
comment.
comment summary variable for provisioninternet (range 0-3.5) = physical provision of internet, 1.5 for good home access abd 1 for poor home access.
comment already added to (max 2) provision above in SECTION B - 0.5 for home 0.5 for work and 1.0 for mobile access.
if b4a=1 provisioninternet=provisioninternet+0.5.
if b4a=1 homeormobileaccess=1.
if b4b=1 provisioninternet=provisioninternet+0.5.
if b4c=1 provisioninternet=provisioninternet+1.0.
if b4c=1 homeormobileaccess=1.
if d1=1 provisioninternet=provisioninternet+1.5.
if d1=2 provisioninternet=provisioninternet+1.
if d1=1 homeormobileaccess=1.
if d1=2 homeormobileaccess=1.
comment PROVISIONHLTH**************************************************************************************.
comment summary variable for provisionhlth=depending on GP and other access.
compute provisionhlth=0.
comment GP d3 website.
if d3=1 provisionhlth=provisionhlth+1.
if (d3=2 and need>0 and need<99) provisionhlth=provisionhlth+0.5.
if (d3=2 and need=0) provisionhlth=provisionhlth+0.75.
if (d3=4 and need=0) provisionhlth=provisionhlth+0.5.
if (d3=4 and need>0 and need<99) provisionhlth=provisionhlth+0.25.
comment D4 repeat prescribing.
if d4=1 provisionhlth=provisionhlth+1.0.
if ((d4=3 or missing (d4)) and need=0) provisionhlth=provisionhlth+0.5.
if ((d4=3 or missing (d4)) and need>0 and need<99) provisionhlth=provisionhlth+0.25.
comment D5 access medical records.
if d5=1 provisionhlth=provisionhlth+1.5.
if ((d5=3 or missing (d5)) and need=0) provisionhlth=provisionhlth+0.5.
if ((d5=3 or missing (d5)) and need>0 and need<99) provisionhlth=provisionhlth+0.25.
comment D6 information from website.
if d6=1 provisionhlth=provisionhlth+0.25.
if (d6=2 or b3a2=1) provisionhlth=provisionhlth+0.75.
comment D7 support from internet.
if d7=1 provisionhlth=provisionhlth+0.25.
if (d7=2 or internethealthcomm=1) provisionhlth=provisionhlth+0.75.
comment*************************************************.
comment usedinternethealth set earlier from B3 or also from question D3, D6, D7 - tried even if not successful.
if internethealthcomm=1 usedinternethealth=1.
if (d6>2 or d7>2 or d3=1) usedinternethealth=1.
compute provision=provisioninternet+provisionhlth.
if missing (b1) provisionhlth=99.
if missing (b1) provisioninternet=99.
```

```
if missing (b1) provision=99.
comment **********************************************************************************************************************.
comment section E.
comment.
comment disability if E1=2 disability=disability+2, if E1=3 disability=disability+1.
if e1=2 disability=disability+2.
if e1=3 disability=disability+1.
comment 99 is missing value, personal not availabel if not used internet.
comment non internet users can score up to 3, internet users start at 3.
comment *********************************************************************************************************************.
comment calculates a SKILLS score to compare with personal rating for those who have accessed Internet.
comment add answers to e2 (=0 if missing), ie does not add anything but not to lose if one subquestion not answered.
compute skills=0.
if e2a>0 skills=skills+e2a.
if e2b>0 skills=skills+e2b.
if e2c>0 skills=skills+e2c.
if e2d>0 skills=skills+e2d.
comment if internet user has skills score and e3 self-effficacy rating.
comment starting point for internet users is 3.
comment personal score for internet users is moderated self-efficacy rating = 3 + personal*skills/12.
if usedinternet=1 personal=3+((e3*7/10-1)*skills/12).
comment************************************************************.
comment section F SUPPORT (Internet users).
comment check for missing data.
if usedinternet=1 support=0.
comment if section not completed support will remain zero, dealt with later in combination of support with personal in readiness.
if f1=1 support=support+2.
if f2=3 support=support+1.5.
if f2xx=1 support=support+1.5.
comment if support has been used it definitely exists and can be found.
comment f4,5 and 6 can contribute 4 to support score.
if f4=2 support=support+1.5.
if f4=3 support=support+0.75.
if f5=1 support=support+1.5.
if f6=1 support=support+1.
comment ********************************************************************************************************************.
comment section G.
comment.
if g1a=4 economic=economic+3.
if g1a=3 economic=economic+2.
if g1a=5 economic=economic+1.
if g1b=4 economic=economic+3.
```

```
if g1b=3 economic=economic+2.
if g1b=5 economic=economic+1.
if g1c=1 economic=economic+1.
if g1d>2 economic=economic+1.
if g1e>2 economic=economic+1.
comment economic variable also has contributions from questions in section D.
comment D1=2 (internet connection is slow) and D2=1 (I would need to pay more) problem is economic.
comment D1=3 (No Internet connection) and D2=1 (I would need to pay more) problem is economic.
comment possible that by reducing economi can go less than zero so needs to be corrected.
if d1=2 and d2=1 economic=economic-1.
if d1=3 and d2=1 economic=economic-1.
if economic<0 economic=0.
if missing(g1a) and missing (g1b) and missing (g1c) and missing (g1d) and missing (g1e) economic=99.
comment set economic to missing if section g not answered at all.
comment go with those answers that have been given if partially answered.
comment *******************************************************************************************************.
comment calculate 'short scores'  to 5 point scores.
compute economic5=rnd (economic/2).
compute provision5= rnd (provision/2).
compute personal5= rnd (personal/2).
compute support5= rnd (support/2).
compute modsupport=0.
if ((support+personal)>0) modsupport=3*support/(personal+support).
compute rndmodsupport=rnd(modsupport).
compute readiness=(personal+provision+modsupport+economic5)*9/24.5.
compute readiness=rnd(readiness).
comment original attempt to combine scores using participant preference to give more weight to factor
rated most important according to answer to H! (overall).
comment previous calculations including weighting from question H1.
comment if H1 not answered or =1 (no need for health information) just sum to get readiness score.
comment if (h1=2 or h1=4) readiness = rnd (((personal*2)+provision+support+economic)/5)..
comment if h1=3 readiness = rnd ((personal + (provision*2) + support + economic)/5).
comment if h1=5 readiness = rnd ((personal + provision + (support*2) + economic)/5).
comment if h1=6 readiness=rnd ((personal + provision + support + (economic*2))/5).
comment if (h1=1) or missing (h1) or (h1=7) readiness= rnd ((personal+provision+support+economic)/
```

6. DEALING WITH INCONSISTENT AND MISSING DATA

There were 16/344 (4.7%) ‘problem’ questionnaires associated with question B1 and its role in directing people to section C or remaining with section B (Query codes: 7,8,9 (shaded)). Many of these needed to have their data ‘corrected’ based on the most likely meaning. Mostly these corrections were simple, for example, (query 8) where people have used the Internet but still answered section C. In that case section C was ‘blanked’. Some were more difficult to interpret, for example, one person (ID 578, male aged 27) who had been a frequent user of the Internet, had a home computer and a smart phone, had used the Internet for a range of purposes, but seemed not to have used the Internet in the last three months. In question H2 he suggested more assistance would help him use the Internet.

Query code	When query applies and what it means (see questionnaire for question numbers).	Respondents with this query
1	a3e=1 and b1=2<br>If respondent has used Internet in last 3 months for health (A3) but ticked no to use it personally in last 3 months on B1. This is possible if they got somebody else to use the Internet for them.	1
2	b1=1 and missing (b2)<br>If respondent has answered that using Internet but then not entered how often.	0
3	b4a=1 and (missing (d1) or d1>2)<br>If b4a=1 (used Internet at home) d1 should =1 or 2, if d1=3 it is possible that the respondent has lost internet connection.<br>Missing d1 and d1=4 suggests that respondent does not understand question of 'speed' – records and questionnaires to be checked. Checking questionnaires suggest this to be the case. Wording perhaps needs changing, but added 1.0 to provisioninternet. (See also query 10)	19
4	d1=3 and b4a=1<br>If respondent has no Internet connection in section D but looks as if they do in section B. This might be if people use a mobile at home. Wording probably needs more clarification.	3
5	d1=1 and d2>0<br>If respondent has adequate home Internet access fine (d1) they should not have answered d2.	3
6	d7>1 and b3b2=0 and b3d2=0 and b3e2=0<br>If respondent has tried to contact someone for health (D7), it should have shown up by using email, discussion forum, etc in section B.	0
7	(b1=2 and b2>0) or (b1=2 and (b4a>0 or b4b>0 or b4c>0 or b4d>0 or b4e>0 or b4f>0)) .If respondent says not used Internet but still completed section B. Record needs to be checked to see how to handle data. If section C completed, CORRECTION is to 'blank' the rest of section B.	4
8	(missing(b1) or b1=1) and c1>0<br>If respondent has completed section C but question B1 not answered or B1=1. CORRECTION is to set B1=2.	6
9	(c1>0 OR C2>0 OR C3>0 or c4>0) and b1=1<br>Section C should not be completed by Internet user. If other answers confirm b1, CORRECTION is to blank section C.	6
10	b4a=1 and (missing (d1) or d1>2)	4

	If b4c=1 it seems likely that they use their mobile at home (wording of B4 perhaps needs changing slightly). Added 1.5 to provisioninternet.	
11	Missing (f1) and missing (f2) and missing (f3) and usedinternet=1<br>Section on support not completed for people who used Internet	21

7. REFERENCES

1. Norman CD, Skinner HA: **eHEALS: The eHealth Literacy Scale.** *Journal of Medical Internet Research* 2006, **8.**
2. van Deursen A, van Dijk J: **Internet Skills Performance Tests: Are People Ready for eHealth?** *Journal of Medical Internet Research* 2011, **13.**
